# Progress of Acupuncture Therapy in Diseases Based on Magnetic Resonance Image Studies: A Literature Review

**DOI:** 10.3389/fnhum.2021.694919

**Published:** 2021-08-20

**Authors:** Jinhuan Zhang, Zihan Li, Zhixian Li, Jiaying Li, Qingmao Hu, Jinping Xu, Haibo Yu

**Affiliations:** ^1^Department of Acupuncture, The Fourth Clinical Medical College of Guangzhou University of Chinese Medicine, Shenzhen, China; ^2^Institute of Biomedical and Health Engineering, Shenzhen Institutes of Advanced Technology, Chinese Academy of Sciences, Shenzhen, China; ^3^Chinese Academy of Sciences (CAS) Key Laboratory of Human-Machine Intelligence-Synergy Systems, Shenzhen Institutes of Advanced Technology, Chinese Academy of Sciences, Shenzhen, China; ^4^School of Artificial Intelligence, University of Chinese Academy of Sciences, Beijing, China; ^5^Shenzhen Traditional Chinese Medicine Hospital, Shenzhen, China

**Keywords:** acupuncture, MRI, diseases, mechanism, review

## Abstract

The neural mechanisms of acupuncture are not well-understood. Over the past decades, an increasing number of studies have used MRI to investigate the response of the brain to acupuncture. The current review aims to provide an update on acupuncture therapy in disease. The PubMed, Embase, Web of Science, and Cochrane Library databases were searched from inception to January 31, 2021. Article selection and data extraction were conducted by two review authors. A total of 107 publications about MRI in acupuncture were included, the collective findings of which were as follows: (1) stroke and GB34 (Yanglingquan) are the most studied disease and acupoint. Related studies suggested that the mechanism of acupuncture treatment for stroke may associate with structural and functional plasticity, left and right hemispheres balance, and activation of brain areas related to movement and cognition. GB34 is mainly used in stroke and Parkinson's disease, which mainly activates brain response in the premotor cortex, the supplementary motor area, and the supramarginal gyrus; (2) resting-state functional MRI (rs-fMRI) and functional connectivity (FC) analysis are the most frequently used approaches; (3) estimates of efficacy and brain response to acupuncture depend on the type of sham acupuncture (SA) used for comparison. Brain processing after acupuncture differs between patients and health controls (HC) and occurs mainly in disorder-related areas. Factors that influence the effect of acupuncture include depth of needling, number and locations of acupoints, and *deqi* and expectation effect, each contributing to the brain response. While studies using MRI have increased understanding of the mechanism underlying the effects of acupuncture, there is scope for development in this field. Due to the small sample sizes, heterogeneous study designs, and analytical methods, the results were inconsistent. Further studies with larger sample sizes, careful experimental design, multimodal neuroimaging techniques, and standardized methods should be conducted to better explain the efficacy and specificity of acupuncture, and to prepare for accurate efficacy prediction in the future.

## Introduction

Acupuncture has been practiced in China for more than 3,000 years as a minimally invasive therapeutic modality of traditional Chinese medicine (TCM) (Zhuang et al., 2013). It has gained increasing popularity and acceptance due to its obvious efficacy (Liang and Wu, 2006). Several systematic reviews have indicated that acupuncture therapy may improve symptoms in various diseases, such as depression (Smith et al., 2018), ischemic stroke (Lu et al., 2016), migraine (Da, 2015), functional diarrhea (Guo et al., 2020), and Alzheimer's disease (Huang et al., 2019). However, the mechanisms underlying the efficacy of acupuncture therapy remain unclear, arousing widespread skepticism and attention.

Since the 1970s, many studies using animal models have shown that the effects of acupuncture are related to the integration of the central nervous system (Han, 2011; Xiao et al., 2018). MRI, as advanced visualization and non-invasive brain imaging technique, can provide comprehensive, multiparametric information on brain anatomy and function (Yousaf et al., 2018). It has been widely used to elucidate the functional and structural response to acupuncture (Usichenko et al., 2015; Li et al., 2020a). Thus, revealing the mechanism underlying the effect of acupuncture has become an area of research interest in recent years.

With an increasing amount of MRI-based research on acupuncture, a summary to date is useful as a basis for further exploration in the future. Although four reviews (He et al., 2015; Scheffold et al., 2015; Cai et al., 2018; Huang et al., 2019) have been conducted on functional MRI (fMRI) studies to explore the mechanism of acupuncture, several limitations are remaining. First, the literature included studies published from 1999 to 2016. However, numerous MRI studies on acupuncture have emerged in recent years and need to be updated. Second, many of the previous studies involved acupuncture in healthy people, but research has shown that acupuncture effects are more apparent in pathological conditions (Han et al., 2019). Moreover, the emergence of acupuncture as a treatment stems from its function in patients rather than healthy people. Third, it may be necessary to summarize the current literature from different perspectives, such as analytic methods and study designs.

Overall, reviews to date on MRI research in acupuncture provide limited understanding of treatment effects in disease. Therefore, the purpose of this study is to provide an updated review of MRI studies on the mechanism of acupuncture therapy in disease, focusing on disease types and acupoints, experimental design and analysis methods, and research topics.

## Materials and Methods

### Literature Search and Study Selection

A systematic search was conducted to find potentially eligible studies published in English from inception to January 2021 in PubMed, EMBASE, Web of Science, and Cochrane Library databases. Keywords were: (1) MRI, blood oxygen level dependent (BOLD), regional homogeneity (ReHo), the amplitude of low-frequency fluctuation (ALFF), fractional ALFF (fALFF), white matter, voxel-based analysis, voxel based morphometry (VBM), Freesurfer, surface-based morphometry, cortical thickness, surface area, cortical volume, gray matter volume, gray matter density, and (2) acupuncture therapy, acupuncture, acupuncture point, ear, body acupuncture, auricular acupuncture, electroacupuncture (EA), and moxibustion. Studies eligible for inclusion met the following criteria: (1) subjects including patients and not only healthy volunteers; (2) the study was an original article that was peer-reviewed and published in English; (3) studies using acupuncture, EA, or laser acupuncture; and (4) subjects underwent two MRI scans before and after acupuncture or one MRI scan during acupuncture. Exclusion criteria were: (1) protocol, case reports, or case series; (2) other interventions that do not belong to traditional acupuncture, such as transcutaneous electrical nerve stimulation, transcutaneous vagus nerve stimulation, and so on; (3) analytical methods using magnetic resonance spectroscopy (MRS); and (4) experimental animal study.

**Figure 1:**
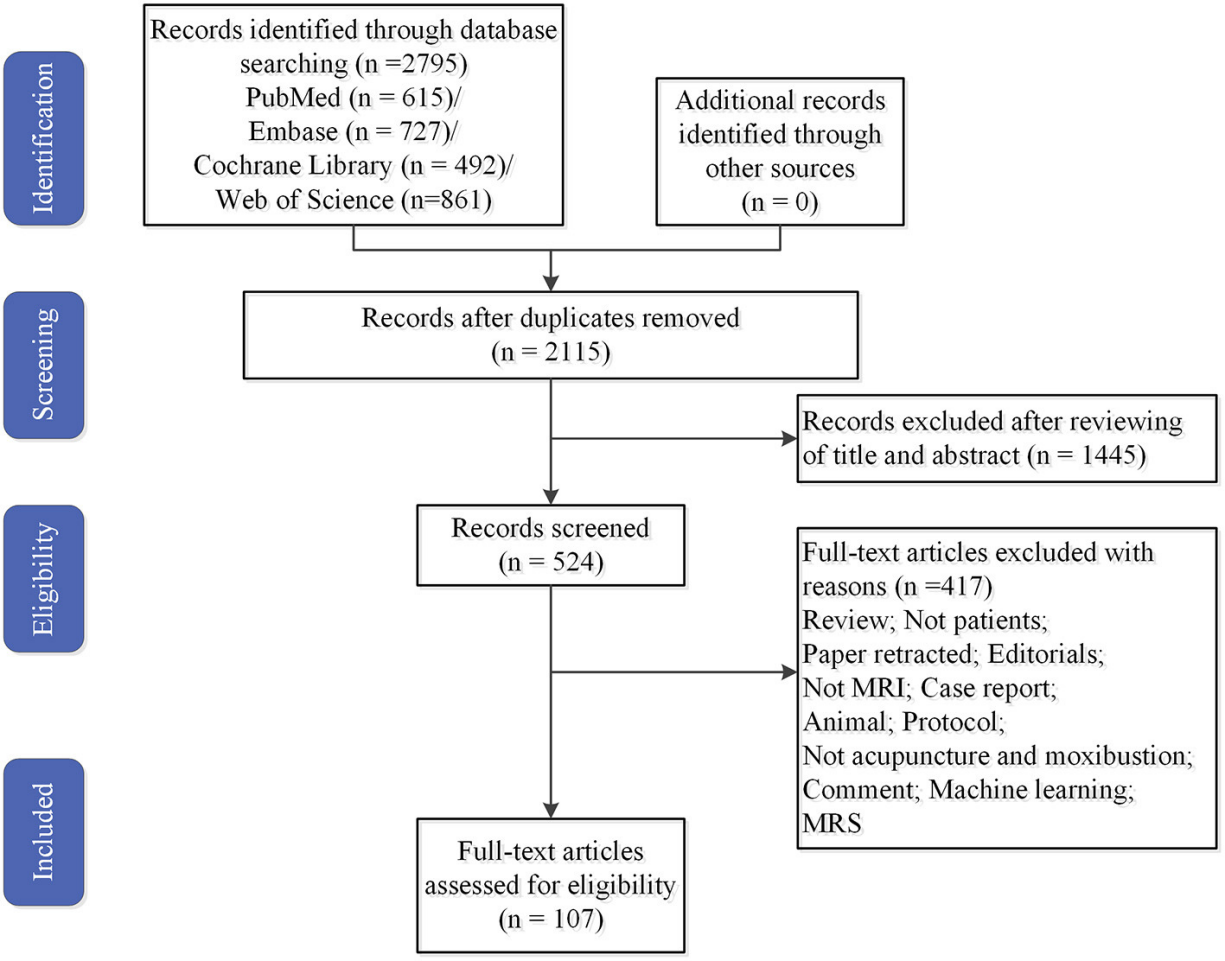
Flowchart of literature selection. MRI, magnetic resonance imaging; MRS, magnetic resonance spectroscopy.

All identified studies were imported into endnote after careful reading of the titles and abstracts. Duplicate studies and those that did not meet the inclusion criteria were excluded. Finally, 107 studies were included (Figure 1).

### Data Extraction

The following data were extracted by two authors: year of publication, author details, number of participants, diseases, intervention/control groups, needling details, types of acupuncture, sample size, acupuncture points, data analysis, experimental design, and study design. Any inconsistencies were discussed with a third author to reach an agreement.

In addition, although several study designs investigating the mechanism of MRI-based acupuncture are involved in this review, considering reliability and rigor of results, we focused in particular on the verum acupuncture (VA) vs. sham acupuncture (SA) in patients and health controls (HC).

## Results

### Study Characteristics

**Figure 2:**
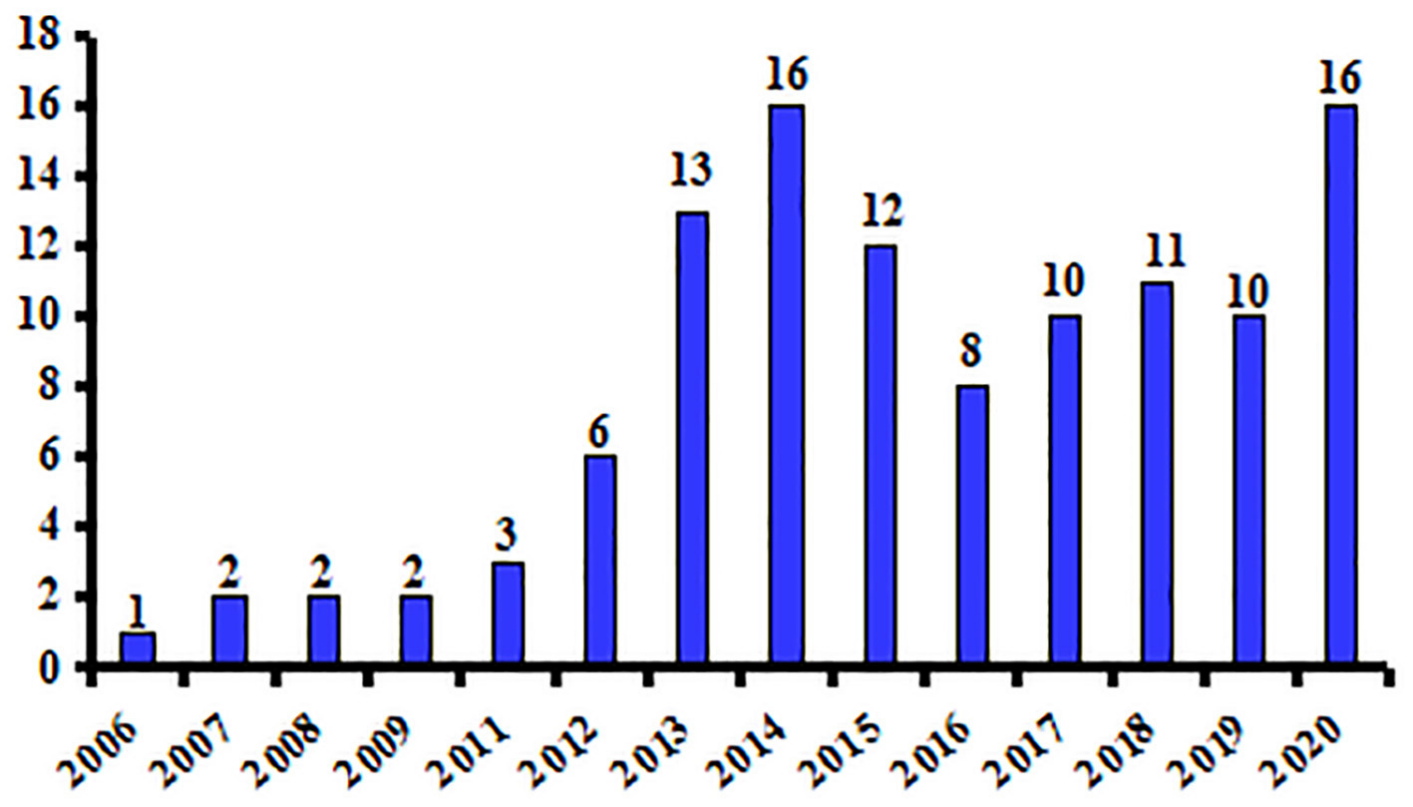
Numbers of MRI studies of acupuncture in diseases.

The search yielded 107 studies that were published between 2006 and 2021 (Supplementary Table 1). The mean sample size was 28 (range 6–102), and the total number of participants in all studies included 2,957 patients and 928 HC. Acupuncture manipulation modality in these studies included manual acupuncture (MA), EA, and laser acupuncture. Figure 2 shows the number of publications per year, with the highest number (*n* = 16) in 2014 and 2020. Seventeen of the studies used 1.5 and 2.0 T MRI systems, and the remaining (85%) used 3.0 T systems.

### Diseases, Acupoints, and Meridians

**Figure 3:**
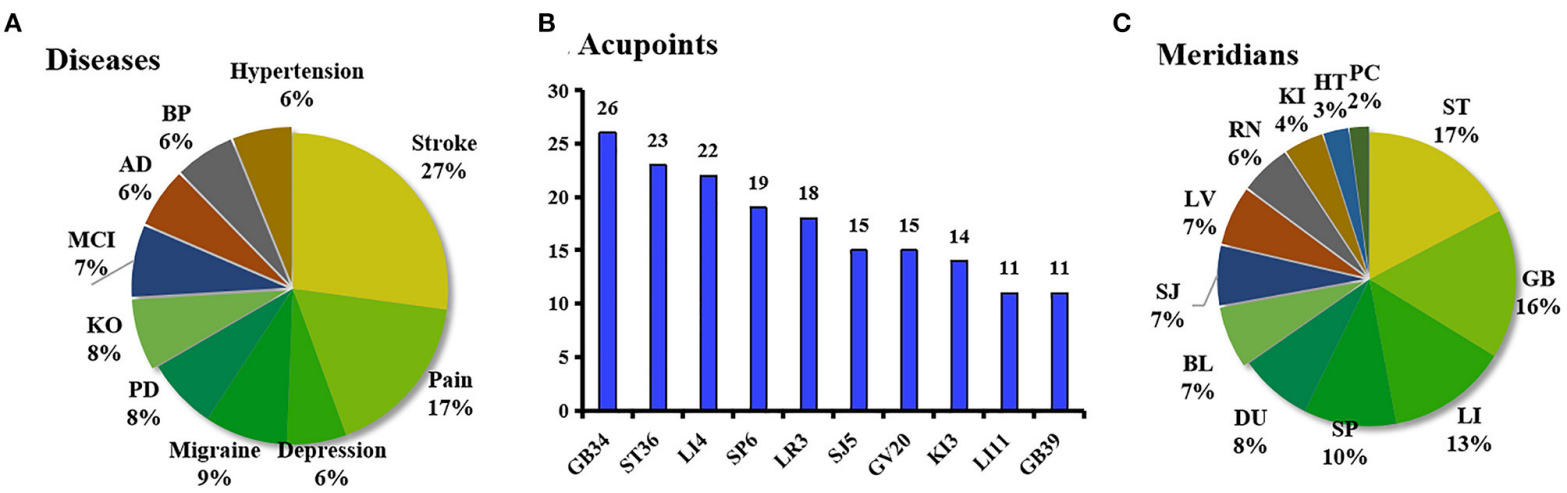
**(A)** Proportion of the top ten most studied diseases in the reviewed studies. **(B)** Frequency of the top ten most used acupoints in the reviewed studies. **(C)** Percentage of meridians where acupoints are located in the reviewed studies. AD, Alzheimer's disease; BP, Bell's palsy; BL, the Bladder Meridian; DU, the Du Meridian; HT, the Heart Meridian; KO, Knee osteoarthritis; LI, the Liver Meridian; MCI, mild cognitive impairment; PD, Parkinson's disease; PC, the Pericardium Meridian; KI, the Kidney Meridian; RN, the Ren Meridian, RN; SJ, the Sanjiao Meridian; SP, the Spleen Meridian.

The studies involved more than 30 types of diseases, mainly related to neurological and digestive systems. The top 10 diseases are shown in Figure 3A. Stroke (including stable somatosensory, chronic, aphasia, ischemic, stable recovery, and subcortical stroke) was the most frequently studied disease.

GB34 (Yanglingquan) was found to be the most frequently applied acupoint and singly applied in 11 studies on stroke (*n* = 8) and Parkinson's disease (*n* = 3). Other acupoints used frequently in the 107 studies are shown in Figure 3B. The meridians used in these studies are summarized in Figure 3C. The acupoints of the stomach meridian in ST36 (Zusanli) were used most commonly, mainly in combination with other acupoints.

### Experimental Designs and Analytic Methods

**Figure 4:**
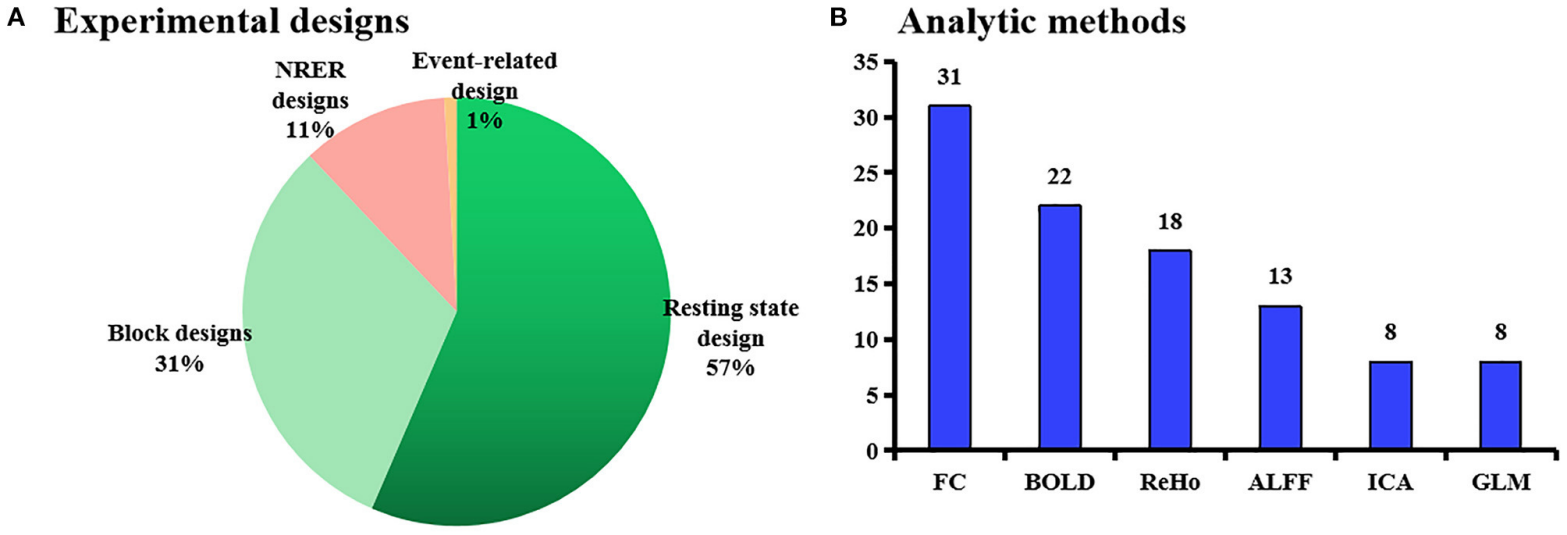
**(A)** Proportion of experimental designs in the reviewed studies. **(B)** Analytical methods used in the reviewed studies. ALFF, the amplitude of low-frequency fluctuation; BOLD, blood oxygen level-dependent; FC, functional connectivity; ICA, independent component analysis; GLM, general linear model, NRER, non-repeated event-related.

Four types of experimental design were applied (Figure 4A), and the resting-state fMRI (rs-fMRI) approach was used most frequently in 60 of the 107 studies. This method was used particularly in the latter 5 years of the publication date range, with 43 of the 60 (70%) being published between 2016 and 2020.

The six most frequently applied analysis methods are summarized in Figure 4B. Functional connectivity (FC) was used most frequently (in 31 studies), whereas VBM and diffusion tensor imaging (DTI) were used less. The earliest application of the FC analysis in this sample was in 2007, but it did not appear for several years. Thirty-one of the studies analyzed FC changes using predefined seed points or regions of interest.

### Research Topics

**Figure 5:**
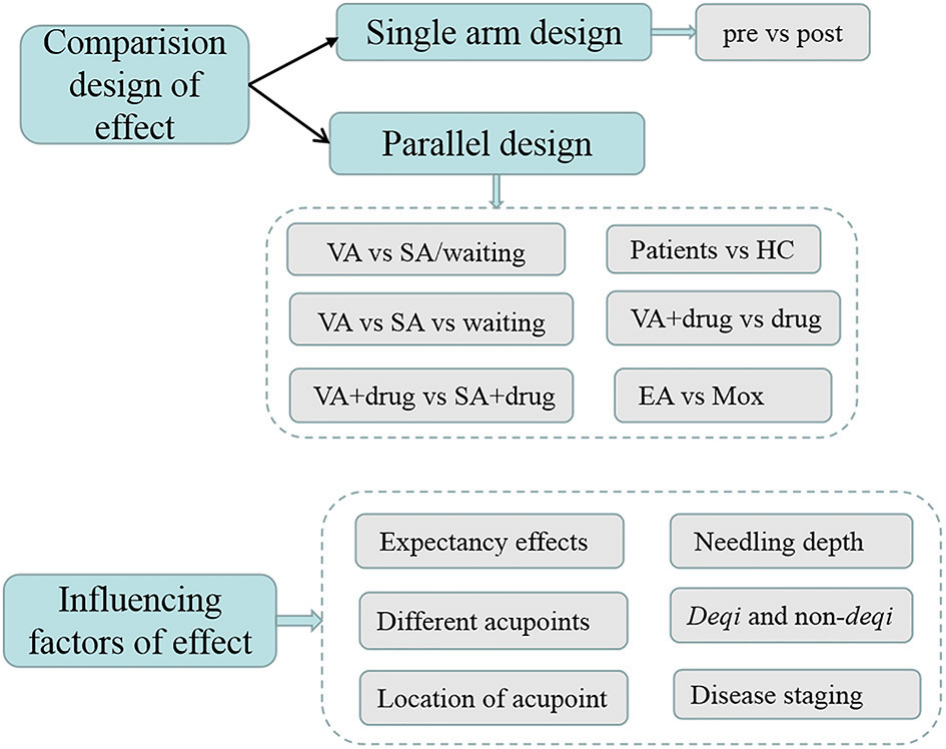
Topics of studies on acupuncture for diseases based on MRI.

The research topics in the included studies could be approximately categorized as MRI investigations of (1) the mechanism by which acupuncture takes effect in disease and (2) factors influencing efficacy (Figure 5).

#### Effect of Acupuncture

Single and parallel arm study designs were used to assess the effect of acupuncture. However, in this review, we focused in particular on the VA vs. SA in patients and HC.

(1) Verum acupuncture and SA
Three types of SA were used in 26 of all studies, including (a) cutaneous stimulation at the acupuncture points or sham points or areas; (b) use of a blunt needle (Streitberger (Streitberger and Kleinhenz, 1998), plastic, or Park (Park et al., 2002) needle: with a blunt tip, to simulate an acupuncture procedure without penetrating the skin); (c) needling at non-acupuncture points close to acupuncture points (Table 1).
  (a) Cutaneous stimulation at the same acupuncture point
  Two studies (Li et al., 2006; Napadow et al., 2007) conducted SA using relatively mild cutaneous stimulation at the same acupuncture point as VA.
  (b) Blunt needles
  A total of eight studies using blunt needles as control showed heterogeneous results. Three studies (Schaechter et al., 2007; Chae et al., 2009; Chen et al., 2015) used the Streitberger needle, one study used the plastic tubes (Wang et al., 2016a), one study used the foam cubes (Chu et al., 2012), and two placebo control studies (Chae et al., 2009; Yeo et al., 2014) used the Park device. All showed significantly higher brain activation in VA than SA, which was related to the pathological mechanism of disease. In addition, two studies (Hashmi et al., 2014; Gollub et al., 2018) using the Streitberger needle investigated the expectation effect in patients with knee osteoarthritis between VA and SA, and found that expectation significantly and similarly modulates the pain experience in both VA and SA.
  (c) Needling at non-acupuncture points close to acupuncture points
  Fourteen (56%) studies (Huang et al., 2011; Liu et al., 2012; Kang et al., 2013; Maeda et al., 2013; Chen et al., 2014; Qi et al., 2014; Zhao et al., 2014; Egorova et al., 2015; von Deneen et al., 2015; Li et al., 2016, 2018; Zheng et al., 2016; Tan et al., 2017; Wang et al., 2020) adapted at non-acupuncture points close to acupuncture points as VA in patients.

**Table 1:** Characteristics of studies about VA and SA.

**Numbers**	**References**	**Subjects**	**Intervention**	**Groups**	**Were HC intervened (Y/N)**	**Acupoints**	**Data analysis**	**Experimental design**	**Type of SA**
**Tactile stimulation**									
1	Li et al., 2006	12 stroke/12 HC	EA	VA/SA	Y	LI4, LI11	SPM	Block/R(45s)-S(45s), 3 times	/
**Blunt needling**									
2	Napadow et al., 2007	13 Carpal Tunnel Syndrome/12 HC	MA	VA/SA	Y	LI4, SJ5, PC7	FC	RS, 3 times/week for 3 weeks and 2 times per week for the remaining 2 weeks.	Non-insertive cutaneous stimulation over the acupoint
3	Schaechter et al., 2007	7 stroke	MA	4 VA/3 SA	/	N	GLM	RS, twice/week for 10 weeks.	Streitberger needle
4	Chae et al., 2009	10 Parkinson's Diseases/10 HC	MA	VA/covert placebo/overt placebo	N	Left GB34	SPM	Block, R(2min)-S(1min), 3 times	Park needle
5	Chen et al., 2015	30 knee osteoarthritis	MA	10 high dose VA/10 low dose VA/10 SA	/	ST35 and Xi yan (extra point), GB34, SP9, GB39 and SP6	FC, left posterior medial prefrontal cortex (pMPFC), Cortical thickness analysis	RS, 6 acupuncture treatment sessions in 1 month (twice/week for the first 2 weeks, once/week for the last 2 weeks	Non-acupoints, Streitberger needle
5	Hashmi et al., 2014	40 knee osteoarthritis	EA	20 VA/20 SA	/	LI3 and LI4	Graph-theoretic metrics of network topology	RS (real or sham acupuncture), lasted 25 min.	Streitberger needle
7	Gollub et al., 2018	43 knee osteoarthritis	MA	21 MA/22 SA	/	Right Liv3 and LI4	ReHo	RS, five times/week, for 4 consecutive weeks.	Streiberger needle
8	Chu et al., 2012	30 irritable bowel syndrome	EA	15 EA/15 SA	/	Bilateral ST36, ST37 and SP6	SPM	Block, 30 min EA	Foam cubes
9	Yeo et al., 2012	12 Parkinson's Disease/12 HC	MA	VA/SA	Y	Right GB34	SPM	R(4min)-sham(S(1min)-R(1min-S(1min)-R(1min))-R(4min)-T1(10min)-verm(S(1min)-R(1min)-S(1min)-R(1min)-R(4min)	The blunt type needle was not inserted into the skin
10	Yeo et al., 2014	12 Parkinson's disease/12 HC	MA	VA/SA	Y	GB34	SPM	Block, SA: R(60s)-S(60s)-R(60s)-S(60s)-R(60s)-structural images(15min); then RA: R(60s)-S(60s)-R(60s)-S(60s)-R(60s)	Park needle
11	Wang et al., 2016b	46 depression	MA	22 MA and fluoxetine/24 SA and fluoxetine	/	RN12, RN10, RN6, RN4, KL 17, ST24, and Qipang.	FC, bilateral amygdala as the seed region	RS, once a day for the first 3 days and subsequently once every 3 days for the remainder of the 8-week trial.	Short plastic tubes
**Needling at non-acupuncture points in close proximity to acupuncture points**									
12	Huang et al., 2011	12 ischemic stroke	MA	6 VA/6 SA	/	SJ5	SPM	Block, S(30s)-R(30sn), 6 times	/
13	Liu et al., 2012	41 myopia	MA	11 VA and TI/ 11 VA and NI/10 SA and TI/9 SA and NI	/	LR3	GLM	RS, R(186s)-S(180s)	/
14	Maeda et al., 2013	59 carpal tunnel syndrome	EA	22 local VA/18 distal VA/19 SA	/	Local VA: PC7, SJ5; distal VA: SP-6, LV-4; SA: SH1 and SH2.	GLM	RS, event-related/2-s stimulation events with randomized interstimulus interval (ISI), 6–12 s, and total scan time 5 min and 6 s	/
15	Kang et al., 2013	25 smoke	MA	12 VA/13 SA	/	Left HT7	SPM	Block, S(60s)-R(60s), 3 times	/
16	Qi et al., 2014	16 ischemic stroke	MA	8 VA/8 SA	/	SJ5	SPM	Block, R(30s)-S(30s), total 6min6s	/
17	Zhao et al., 2014	40 migraine	MA	20 active point/20 inactive point	/	Active point: bilateral SJ5,GB20, GB34, and GB40 inactive point: bilateral SJ22, PC7, GB37, and SP3	ReHo	RS, 4 times/week for 30 min each for 8 weeks	/
18	Chen et al., 2014	6 ischemic stroke	MA	VA/SA	/	SJ5	SPM	Block, R(5 min)-SAstimulus(6 min30s)-R(6mmin2s)-VA(6 min30 s).	/
19	Zhu et al., 2014	28 irritable bowel syndromes	Mox	15 VA/13 SA	/	ST25, RN6, and RN12	ICA	RS, three times/week for 4 weeks	2 cm in diameter, 1.14 g in weight
20	von Deneen et al., 2015	19 overweight	MA	10 MA/9 SA	/	Bilateral ST 36 and SP 9	SPM	Block, R(5 min)-S(21min)-S(1min)-R(7 min) anatomical scan -(9 min) postscan	/
21	Egorova et al., 2015	41 knee osteoarthritis	MA	6 acupoints/2 acupoints/SA	/	2 acupoint: ST35 and Xi yan (extra point) and 6 acupoints: additionally, at GB34, SP9, GB39 and SP6	FC, periaqueductal gray (PAG) as the seed	RS, 6 times 25 min acupuncture sessions in 1 month	/
22	Li et al., 2016	62 migraine/46 HC	MA	11 VA1/11 VA2/13 VA3/11 SA/16 WT	N	VA1: GB34, GB40 and SJ5; VA2: GB33, GB42 and SJ8; VA3: ST36, ST42 -and L16; SA: NAP1, NAP2 and NAP3	FC, periaqueductal gray (PAG) as the seed region	RS, 30 min, 5 times/week, 4 weeks.	/
23	Zheng et al., 2016	28 hypertension	MA	14 MA/14 SA	/	LR3	FC, hypothalamus as the seed	RS, 30 min, three times/week for 2 week	/
24	Tan et al., 2017	32 mild cognitive impairment	MA	16 MA/16 SA	/	EX-HN1, EX-HN3, PC6, KI3, ST40, and LR3	FC, pregenual ACC, sub-ACC, MTL-hippocampus, IPL, and anterior insula as the seed region	RS, 5 times/week, for 4 consecutive weeks.	/
25	Li et al., 2018	35 Parkinson's disease	MA	14 MA/11 SA/10 WT	/	DU20, bilateral GB20, and the Chorea-Tremor Controlled Zone	DC,ReHo,ALFF	RS, 30 min, 2 times/week, for 12 weeks	/
26	Wang et al., 2020	45 primary insomnia	MA	15 S-Acu/15 M-Acu/15 N-Acu	/	S-Acu: bilateral HT7; M-Acu: combination of bilateral HT-7, bilateral SP6 and single DU20; N-Acu: sham point	fALFF	RS, 30 min, 5 times/week, for 5 weeks	/

*ACC, anterior cingulate cortex; DC, degree centrality; EA, Electroacupuncture; fALFF, fractional amplitude of low-frequency fluctuations; FC, functional connectivity analysis; GLM, general linear model; HC, health controls; ICA, independent component analysis; MA, manual acupuncture; M, multiple; /, irrelevant; NAP, non-acupoint; NI, non-treatment instruction; N, no; NRER, non-repeated event-related; RS, resting state; ReHo, regional homogeneity; SA: sham acupuncture; S, single; TI, treatment instruction; WT, waiting list; VA, verum acupuncture; Y, yes; Acupoints: BL23, Shenshu; BL25, Dachangshu; BL40, Weizhong; BL62, Shenmai; BL26, Guanyuanshu; BL36, Chengfu; BL54, Zhibian; BL60, Kunlun; BL58, Feiyang; BL27, Xiaochangshu; BL37, Yinmen; DU3, Yaoyangguan; DU20, Baihui; DU23, Shangxing; DU29, Yintang; EX-HN5,Taiyang; EX-HN1, Sishenchong; GB8, Shuaigu; GB30, Huantiao; GB31, Fengshi; GB33,Xiyangguan; GB39, Xuanzhong; GB40, Qiuxu; GB41, Zulinqi; HT3, Shaohai; HT5, Tongli; HT7, Shenmen; KI3, Taixi; LI3, Sanjian; LI4, Hegu; LI6, Pianli; LI7, Lieque; LR8, Ququan; LR14,Jimen; LR3, Taichong; PC6, Neiguan; PC7, Daling; PC8, Laogong; RN4, Guanyuan; RN6,Qihai; RN10, Xiawan; RN12, Zhongwan;RN14, Juque; SJ6, Zhigou; SJ5, Waiguan; SJ8, Sanyangluo; SJ22, Erheliao; SJ23, Sizhukong; SP6, Sanyinjiao; SP8, Diji; SP9, Yinlingquan; SP3, Taibai; SP14, Fujie; SP11, Jimen; SP13, Fushe; SP15, Daheng; ST6, Jiache; ST9, Renying; ST21,Liangmen; ST24, Huaroumen; ST25, Tianshu; ST26, Wailing; ST27, Daju; ST35, Dubi; ST36, Zusanli; ST37, Shangjuxu; ST38, Tiaokou; ST39, Xiajuxu; ST40, Fenglong; ST42, Chongyang; ST44, Neiting*.

Overall, there was a great deal of heterogeneity between the studies. Some studies (Li et al., 2016; Wang et al., 2020) showed no statistical and clinical significance between the real acupoint group and the non-acupoint group. Other studies (Tan et al., 2017; Li et al., 2018) showed significant improvements in clinical symptoms, however, needling produced more significant activation at acupuncture points than at non-acupoints.

Interestingly, two studies (Huang et al., 2011; Chen et al., 2014) randomly assigned stroke patients to two groups: one group underwent sham needling (tactile stimulation) and true needling at the SJ5 (Waiguan) in healthy upper limb and the other group underwent sham and true needling at a sham point. Results showed that needling at SJ5 in healthy upper limbs of stroke patients resulted in reduced activation of brain functional areas, with no evident activation points, compared with tactile stimulation and needling at sham points. In addition, a significant difference in activation reduction was found between tactile stimulation and needling in sham point groups.

(2) Patients and HC
A total of 20 studies on neural response of patients and HC to acupuncture were included (Table 2).

**Table 2:** Characteristics of studies about patients and HC.

**Numbers**	**References**	**Subjects**	**Intervention**	**Groups**	**Acupoints**	**Data analysis**	**Experimental design**	**Results**
1	Li et al., 2006	12 stroke/12 HC	EA	/	LI4 and LI11	SPM	Block/R(45s)-S(45s), 3 times	Compared to HC, patients showed greater activation in the somatosensory cortex with both the tactile task and the acupoint stimulation
2	Wu et al., 2008	11 spastic cerebral palsy/10 HC	MA	/	Left LI3	SPM	Block/S(1min)-R(1min),3 times	Compared to HC, the children with cerebral showed significant decrease in the frontal lobe contralateral temporal lobe, and parahippocampal gyrus; and signal increase in bilateral occipitallobe and ipsilateral insula.
3	Li and Yang, 2011	7 aphasia stroke/14 HC	EA	/	SJ 8	SPM	Block/R(45s)-S(45s), 3 times	Compared to HC, patients showed significant activation in the opercular, triangular, or insula.
4	Feng et al., 2012	12 mild cognitive impairment/12 HC	MA	DA/SA	KI3	FC	NRER/R(1min)-S(DA/SA)(2min)-R(6min)	Compared to HC, mild cognitive impairment patients showed enhanced the correlations related with the temporal regions.
5	Yeo et al., 2012	12 Parkinson's Disease/12 HC	MA	VA/SA	Right GB34	SPM	R(4min)-sham(S(1min)-R(1min-S(1min)-R(1min)-R(4min)-T1(10min)-verm(S(1min)-R(1min)-S(1min)-R(1min)-R(4min)	Compared to the HC, patients with Parkinson's disease showed a significantly higher signal increase in the thalamus
6	Chen J. et al., 2013	10 ischemic stroke/6 HC	MA	/	SJ5	SPM	Block/R(30s)-S(30s), 6 times	Compared to the HC, stroke patients showed enhanced activation in the left Brodmann areas, hypothalamus, and the ventral posterolateral nucleus, and the right BA4, 6, 7, 18, 19, and 32.
7	Cho et al., 2013	11 stroke/10 HC	MA	/	LI11 and ST36	SPM	Block/R(30s)-S(30s), 3 times	Compared to the HC, stroke group showed less brain activation.
8	Zhou et al., 2013	24 functional diarrhea/24 HC	MA	/	ST25	ReHo and FC	RS/2 weeks, 5 sessions per week	Compared to HC, functional diarrhea was more effective in clinical outcome and had more extensive cerebral ReHo changes.
9	Quah-Smith et al., 2013	10 depression/10 HC	LA	/	LR14, LR8, RN14, and HT7	ICA	Task/block(2s delay-R(2s)-s(20s))/8 times, 8 session	Compared to HC, depressed participants had wider posterior default mode network modulation at the parieto–temporal–limbic cortices.
10	Zhang et al., 2014	8 ischemic stroke/10 HC	MA	DA/SA	GB34	SPM	NRER/R(1min)-S(1min)-R(8min)	Compared to HC, stroke patients showed the enhanced interregional interactions between the anterior cingulate cortex (ACC) and posterior cingulate cortex (PCC).
11	Xie et al., 2014	9 stable recovery stroke/8 HC	MA	/	GB34	GLM and GCA	NRER/R(1min)-S(1min)-R(8min)	Compared to HC, stroke patients showed specific modulations of motor-related network in stroke patients.
12	He et al., 2014	28 Bell's palsy/20 HC	MA	18 early group/21 late group/19 recovered group	LI4 on the contralateral side of paresis	FC, SI as the seed region	RS/10 min, three times a week	No significant changes in HC, Bell's pals patients showed significant connectivity changes in the SI region.
13	Wang et al., 2014	14 Alzheimer's Disease/14 HC	MA	/	Bilateral LR3 and LI4	FC, bilateral hippocampus as the seed region	Block/R(3min)-S(3min)-R(10min)	No significant changes in HC, Alzheimer's Disease(AD) patients showed increased FC in right hippocampus and right STG.
14	Liang et al., 2014	9 Alzheimer's Disease/11 HC	MA	/	Right Liv3 and LI4	ICA	Block/R(3min)-S(3min)-R(10min)	All the brain activation modulated by acupuncture were specifically for AD but not for HC.
15	Li et al., 2014d	24 functional dyspepsia/24 HC	MA	/	Right ST36	GLM	Block/R(1min)-S(1min)-R(1min)-S(1min)-R(1min)-S(1min)-R(1min)	Compared to HC, acupuncture evoked pronounced changes, especially in the homeostatic afferent processing network of FD patients.
16	Yeo et al., 2014	12 Parkinson's disease/12 HC	MA	VA/SA	GB34	SPM	Block/SA:R(60s)-S(60s)-R(60s)-S(60s)-R(60s)-structural images(15min); then RA:R(60s)-S(60s)-R(60s)-S(60s)-R(60s)	Compared with HC, patients with PD showed significantly higher brain activity in the prefrontal cortex and precentral gyrus, especially visible in the left hemisphere. which are all known to be affected by PD
17	Gao et al., 2015	10 ischemic stroke/10 HC	MA	/	ST36	SPM	Block/(30s)-S(30s), 6 times	Significant regions in the HC included the prefrontal cortex, cingulum, thalamus, and cerebellum; Significant regions in the stroke patients included the cuneus, supplementary motor area, and inferior parietal gyrus.
18	Wu et al., 2015	20 Bell's Palsy/20 HC	MA	/	LI4	FC, ACC as the seed region	RS, block/R(10min)-S(10min, Rotate needles10 s every 2 min)-R(10min)	Significant decreased connectivity of the right ACC in the early group, no significant effect on FC of bilateral ACC in HC.
19	Han et al., 2019	22 poststroke motor impairment/22 HC	MA	/	GB34	TBSS and FC	NRER/R(8 min10s)-S(1min)-R(8 min10s)	Compared with the HC, stroke patients showed enhanced FC between the (PM)/supplementary motor area (SMA) and supramarginal gyrus (SMG).
20	Han et al., 2020	26 ischemic stroke/21 HC	MA	/	GB34	Graph Theoretical Network Analysis	RS/R(8min10s)-S(60s)-R(8min10s)	Compared with the HC, the stroke patients had a decreased normalized small-worldness (σ), global efficiency (Eg), and the mean local efficiency (Eloc) of the whole-brain network in the resting state.

*LA, laser acupuncture; SI, primary somatosensory area; TBSS, tract-based spatial statistics*.

Except for three studies (He et al., 2014; Liang et al., 2014; Wang et al., 2014) that showed no significant brain activation in HC, most found that similar brain regions are activated by acupuncture in patients and HC. However, in patients, brain region activation and enhanced FC caused by acupuncture were disease-related brain areas.

#### Factors Influencing the Effect of Acupuncture

The factors influencing acupuncture efficacy have always been of strong research interest, which are helpful to increase understanding of the therapeutic mechanism and to provide guidance for clinical practice. However, it is not clear how these factors affect clinical efficacy.

Factors identified as potential influencing acupuncture efficacy are shown in Supplementary Table 2. Several studies investigated the influence of expectation, treatment instruction, augmented context, duration of acupuncture, disease stage, and selection of acupoints on the effect of acupuncture and the related brain response.

(a) Needling depth
Three studies (Feng et al., 2012; Bai et al., 2013; Chen S. et al., 2013) were performed to compare the effects and brain responses between deep and shallow needling, and results showed that deep needling is relatively more effective in mild cognitive impairment.
(b) *Deqi* and non-*deqi*
Two studies were performed to explore the differences in brain function between *deqi* and non-*deqi*, and results showed stronger brain activation in the *deqi* group.
(c) Expectation effects
Five studies (Liu et al., 2012; Hashmi et al., 2014; Gollub et al., 2018; Kong et al., 2018; Tu et al., 2019) were performed to explore the expectation effect, and results showed that expectation reduced symptoms and stimulated brain activity, which was influenced by the form of expectation.
(d) Selection of different acupoints
Three studies (Wang et al., 2016b, 2020; Zhang et al., 2020) were performed to compare the effects and brain mechanisms between single and multiple acupoints on diseases, mainly related to hypertension and primary insomnia. The results consistently showed that a combination of multiple acupoints was more effective by activating more brain regions than single acupoints.

In addition, several studies (Chen et al., 2015; Egorova et al., 2015; Li et al., 2016, 2017, 2020b) were performed to compare different acupoints or dosage levels, and results reported clinical but not brain activation differences, which might be due to small sample sizes. The consensus of these findings was that a combination of different acupoints has no significant difference in treatment effect.

(e) Location of acupoint
  ① Ipsilateral and contralateral sides [opposing needling (ON)]
  Two fMRI studies (Zhang et al., 2018; Yan et al., 2020) on unilateral chronic shoulder pain compared brain activation in acupuncture on ipsilateral and contralateral sides (ON). They found that treatment on either side alleviates pain intensity and improves shoulder function, but the latter improvement was higher in the contralateral than the ipsilateral group. In addition, ReHo values and degree centrality (DC) differed between these groups.
  ② Local point and distal point
  One study (Maeda et al., 2013) was performed to compare the therapeutic effects of and brain response to acupuncture at local and distal points and found that visual analog scores for paresthesia showed significant reductions in the local but not distal group. In terms of brain response, consistent activation in two groups was found in the bilateral insula and secondary somatosensory cortex.

## Discussions

Research on TCM has reached a point at which researchers should pause and reflect on its future directions. For thousands of years, its curative effect has not been widely accepted because it was based on unclear mechanisms. Therefore, a relatively objective theoretical system needs to be formed in the future for the sake of long-term development. The meridian effect was one of the essential building blocks of TCM theory and acupuncture. In recent years, the specificity of meridian acupoints has also become a research focus (Rong et al., 2013; Li et al., 2014a). Such a progression of research trends is in line with the development of modern technology such as fMRI, which was not available in the past.

To enhance understanding of the mechanism of acupuncture revealed by MRI, we conducted a comprehensive literature search with three key findings: (1) stroke and GB34 were the most studied disease and acupoint, respectively; (2) rs-fMRI and FC were the most often used experimental and analytic methods; (3) despite the heterogeneity among studies, the general trend was that effects are more specific in VA than SA, and that brain activation effects of needling inpatients were more specific than in HC. In addition, factors affecting the efficacy of acupuncture mainly included depth, acupoint, *deqi*, location, and expectancy effects.

### Diseases and Acupoints

The most common disease types considered in the included studies were neuropsychiatric, perhaps due to the known treatment efficacy of acupuncture in these diseases. Among these, stroke was the most frequently studied. Stroke, defined as a neurological deficit attributed to an acute focal vascular injury of the central nervous system (Sacco et al., 2013), is a major cause of death and disability globally (Campbell and Khatri, 2020). It brings burdens to the family, seriously affecting the quality of life of the patient (Dowswell et al., 2000) which may be improved by rehabilitation of total motor dysfunction (Hamzat and Peters, 2009).

Acupuncture is recommended by the WHO as an alternative and complementary strategy for stroke treatment (Chavez et al., 2017). Clinical trial and meta-analysis findings have demonstrated the efficacy of acupuncture in improving balance function, reducing spasticity, and increasing muscle strength and general well-being post-stroke (Liu et al., 2009; Zhao et al., 2009; Chavez et al., 2017). Thus, uncovering the mechanism of the effect of acupuncture on movement in stroke has been of strong research interest in recent years.

Studies have found that acupuncture could not only induce brain activation in the motor and sensorimotor networks and increase motor-cognition connectivity but also enhance FC between the bilateral primary motor cortices and the default mode network (Ning et al., 2017). In addition, acupuncture could evoke pronounced structural reorganization (Wu et al., 2018). Importantly, research has shown that improvements in function and structure are interrelated, structural plasticity being associated with recovery of motor ability. However, due to small sample sizes and differences in affected brain regions, study designs and analytic methods, the precise link between acupoint, disease, and brain region remains unclear.

With the exception of GV20 (Baihui), the most frequently used acupoints in the included studies were in the four limbs, since these locations are accessible in the scanning state.

GB34 is located on the fibular aspect of the leg in the depression anterior and distal to the head of the fibula. According to TCM theory, GB34 was not only the “he” (meeting) point of the Gallbladder Meridian of Foot-Shao yang but also the influential point of tendons. Therefore, GB34 is often used to treat diseases of the motor system in clinical practice and trials, for example, recovering motor function for patients with stroke hemiplegia (Fang et al., 2016; Yang et al., 2016). In addition, different acupuncture techniques at GB34, such as MA, EA, fire acupuncture, and moxibustion, showed specific characteristics of therapeutic effects. Specifically, the main roles of moxibustion at GB34 are to warm the meridians, regulate Qi, and promote blood circulation, to relieve pain, EA at GB34 mainly focused on treating the motor system, and fire acupuncture at GB34 showed both above effects. One recent review (Xiaoling et al., 2020) investigated fMRI in acupuncture at GB34 and found activation of specific brain areas in the bilateral superior temporal gyrus, bilateral anterior central gyrus, bilateral orbital gyrus, and right inferior temporal gyrus. However, high heterogeneity and conflicting results were found among studies, indicating that further exploration is necessary based on large samples and careful experimental design to understand how to obtain reliable and stable brain responses to acupuncture at GB34.

### Experimental Designs and Analytic Methods

Task state-fMRI during acupuncture administrations is often used to observe the immediate effect of acupuncture. Scanning methods are mostly blocked design and non-repeat event-related design, which can obtain real-time imaging data of the brain regions activated or inhibited by acupuncture, and signal to the noise level of BOLD responses may be increased by manipulating the acupuncture stimulation (Lee et al., 2016; Yin et al., 2019).

Although this method is simple and easy to perform, the experimental period is short. According to the theory of TCM, the effect of acupuncture may depend on the cumulative effect, which has been confirmed by several studies (Shi et al., 2012; Li et al., 2014b). Thus, it may be more suitable to explore the mechanism of immediate effect. The task state-fMRI design can be used to explore the specificity of different acupoints, acupuncture manipulation, and the response of different diseases to the same acupoints. In addition, this design is very suitable for diseases that respond quickly to acupuncture, such as pain diseases (Shi et al., 2015; Zhang et al., 2018). The rs-fMRI technique is a relatively novel approach in which participants are typically asked to rest quietly with their eyes open or closed for several minutes without performing any task (Mwansisya et al., 2017). Rs-fMRI investigates naturally occurring low-frequency (typically 0.01–0.08 Hz) fluctuations in BOLD signals, which has been considered to reflect physiologically meaningful changes of spontaneous neural activity in the resting-state networks (Mwansisya et al., 2017; Takamura and Hanakawa, 2017). Moreover, the network system in the resting state has a considerable degree of stability and presents a high degree of FC (Greicius et al., 2003). More importantly, it is well-known that acupuncture has a cumulative effect. This kind of experimental design is used to observe the changes in brain response after acupuncture treatment, which is helpful to explore the mechanism of long-term acupuncture effect such as investigating the effect of different courses of acupuncture and among different patients, and it is suitable for chronic diseases, such as insomnia (Wang et al., 2020) and depression (Wang et al., 2016b).

Various methods have been proposed for the processing and analysis of MRI data including structural MRI, Rs-fMRI, and DTI. In the present review, structural MRI, which plays an important role to help in understanding the anatomical changes related to acupuncture, mainly involved VBM and surfaced-based morphometry, whereas rs-fMRI mainly involved ReHo, ALFF, FC, seed-based correlation analysis, independent component analysis, and graph-theoretic metrics (Lee et al., 2013; Smitha et al., 2017), which have been widely used to gain a greater understanding of brain circuitry changes after acupuncture.

Different analytical methods are used to investigate the efficacy of acupuncture from different perspectives. FC, which is most commonly used, is defined as the temporal correlation between spatially remote neurophysiological events (Fu et al., 2017), expressed as deviation from statistical independence (temporal correlation) across these events in distributed neuronal groups and areas (Fingelkurts et al., 2005). This may suggest that neural plasticity may potentially be a bridge between acupuncture and the treatment of various diseases such as stroke.

Most studies used seed-based FC due to simplicity and ease of interpretation, with the advantage of focusing only on specific brain regions of interest and not the entire brain network. However, the resulting FC network is dependent on the selection of seed location, and the final FC network may vary significantly even if the seed location changes slightly (Cole et al., 2010; Sohn et al., 2015). Thus, the FC network is highly dependent on seed choice, leading to variation that may obscure results (Bell et al., 2019). Based on the above information, the selection of seed points and the combination of a variety of analytical methods are still important. As for the changes of FC caused by acupuncture, it is equally important to select the appropriate seed point to investigate the mechanism of acupuncture for different diseases.

Interestingly, few studies have focused on structural MRI analysis, perhaps due to the fact that structural change is relatively hard to observe, especially for immediate or short-term effects. While many previous studies reported no significant structural changes, the possibility that they occur could not be excluded, and more studies using modern methods are warranted for verification. Indeed, multimodal MRI may enhance understanding of the mechanism underpinning acupuncture effects due to the well-established links between structure and function of the brain.

### Effect of Acupuncture

#### Verum Acupuncture and SA

Despite over 3,500 clinical studies on acupuncture, the debate about its effect continues (Colquhoun and Novella, 2013). Imaging technology provides an objective basis for the difference in curative effect between VA and SA. Consistent with several previous studies (Yoo et al., 2007; Deng et al., 2008; Dougherty et al., 2008), we found that VA significantly improves clinical symptoms and brain activation related to disease.

However, previous findings in HC are inconsistent. Some studies provided showed evidence that acupoints may have their functional specificity (He et al., 2015), whereas other findings (Fang et al., 2009; Hui et al., 2009) showed that no statistical difference was found between acupoints and sham acupoints. This may be explained by similar segmental innervations in HC. As shown by a recent systematic review (Ots et al., 2020), VA and SA have similar therapeutic effects due to their position in overlapping dermatomes, which partly explain several previous clinical studies (Assefi et al., 2005; Hinman et al., 2014) that showed no significant difference in the effect of VA compared with SA. Thus, to obtain reliable results, the location of the needles selected for SA should be at non-overlapping cutaneous segments based on the knowledge of segmental anatomy.

In addition, acupuncture at different acupoints could achieve different brain activation through dynamic reconstruction of neural networks and, thus, achieve therapeutic effects (Qin et al., 2011). However, Cho et al. (2006) found that acupoint and sham acupoint (away from the Meridians) showed a striking similarity in fMRI results, indicating that acupuncture is effective in pain relief regardless of the choice of point. Moreover, the authors proposed that the effect of acupuncture was one of stress analgesia alone. An activation likelihood estimation meta-analysis of fMRI studies (Chae et al., 2013) showed that a similar but weaker pattern of response was observed with control tactile stimulation than with acupuncture needling. Previous studies (Treede et al., 1999; Kong et al., 2010) also found that the brain areas in which activation was changed by acupuncture needle stimulation largely overlap with those that constitute the so-called pain matrix, suggesting that the brain response to acupuncture is triggered by the pain of needle penetration into the skin. Neuroimaging data demonstrate that placebo analgesia activates subcortical and cortical opioid sensitive brain regions, such as the periaqueductal gray, rostral anterior cingulate, and thalamus, many of which overlap with the area of acupuncture modulation (Dhond et al., 2007). It is important to note that the above studies all included patients with pain, and the placebo response is often strong in pain studies (Vase and Wartolowska, 2019).

In summary, brain activation may be observed in SA and responses may be similar to those of VA in both patients and HC. The type of disease and the acupoints used should be considered when deciding which type of SA to be used as control.

#### Patients and HC

In this review, we included 20 studies that investigated differences in acupuncture-related brain activation in patients and HC. Differences in brain activation between these groups indicated higher specificity in patients than HC in brain regions associated with the relevant disease. These findings imply functional specificity of acupuncture, indicating that acupuncture exerted obvious effects under a pathological condition (Han et al., 2019).

To date, research has shown that the therapeutic effects of acupuncture are achieved not by relieving the diseased area locally but by reestablishing the balance of the internal milieu (involving Ying/Yang, the Five Elements, and the Zang-Fus) (Leung, 2012). It is generally agreed that acupuncture plays a homeostatic role, and thus may have a greater effect on patients with a pathological imbalance compared to HC (Kaptchuk, 2002). Therefore, a lack of significant effect in fMRI studies on healthy subjects may reflect homeostasis existing prior to the intervention in those individuals.

Based on the TCM theory, acupuncture could regulate the body in a bidirectional manner, the regulatory effects differing under physiological and pathological conditions. Acupuncture is a normal part of physiological functions for healthy people and a reflection of the normal Qi and blood running conditions. However, for patients, therapeutic effects of acupuncture are observed in disease, under pathological conditions. Interestingly, one meta-analysis showed that experimental pain in HC and chronic clinical pain conditions in patients have overlapping brain activation patterns, but the mechanism differs (Apkarian et al., 2005).

Thus, more scientific evidence is needed to convincingly demonstrate the specificity of acupuncture in patients.

### Factors Influencing Efficacy

#### Depth of Acupuncture

Acupuncture textbooks provide recommended ranges for the depth of insertion, mainly for safety purposes (Maoliang and Shanchen, 1985). In this review, the depth of insertion is considered as a factor in the efficacy of acupuncture. However, MacPherson et al. (2008) found no significant difference in the activation of brain regions between superficial and deep acupuncture in healthy subjects, perhaps related to the used acupoint (LI4, Hegu), where *deqi* and efficacy are easily achieved.

In this review, studies investigated the efficacy and mechanism of acupuncture for mild cognitive impairment at different depths using different analytic methods, and they found that deep acupuncture is necessary to achieve significant clinical results (Feng et al., 2012; Bai et al., 2013; Chen S. et al., 2013). Such findings are consistent with the Layer Analysis as described in the Yellow Emperor's Inner Classic (Goh et al., 2014). Moreover, the studies consistently showed that deep needling affected a larger number of abnormal brain regions than superficial needling, especially at the hippocampus. In addition, deep needling can induce much stronger and wider-ranging *deqi* (Bai et al., 2013).

Physiological mechanisms related to the effect of deep insertion can be explained based on the structures affected by the needle: the skin, muscle fascia, and muscle. At greater depths, needling may better interact with ascending nerve tracks than with cutaneous afferents (Goldman et al., 2010). Sandberg et al. (2003) indicated that the intensity of *deqi* resulted in a pronounced increase in both skin and muscle blood flows using photoplethysmography, demonstrating that the depth of acupuncture also is an important factor for *deqi*.

#### Deqi

For decades, it has been thought that the *deqi* of acupuncture is related to clinical efficacy. Several studies (Li et al., 2014c; Shi et al., 2014; Yin et al., 2017; Zhao et al., 2017) reported that *deqi* group can significantly reduce the severity of symptoms and showed better efficacy compared with acupuncture without *deqi*. *Deqi* in pain may be driven by slow conduction of pain fibers. Integrating these signals into the central nervous system leads to the modulation of other sensory inputs, which is at least part of the acupuncture effects (Zhou and Benharash, 2014).

Results from two studies (Li et al., 2015; Sun et al., 2020) on acupuncture for a pathological condition were inconsistent with previous research (Sun et al., 2013) on *deqi*, showing that the effect of *deqi* was related to the processing of somatosensory or pain signals. The discrepancy between results was explained by small sample sizes, different physiological states, diseases, and acupoints.

Although we are aware of the correlation between *deqi* and clinical efficacy, as described by Sun et al. (2013), standardization of the quantitative methods of *deqi*, deeper understanding of the link between *deqi* and sharp pain, and improvements of statistical methods are necessary to better investigate the mechanism of *deqi*.

#### Expectation

Studies suggested that non-specific factors such as the expectations of a participant could significantly modulate the effects of acupuncture treatment (Pariente et al., 2005; Kong et al., 2009) and play an important role in the placebo response (Kaptchuk et al., 2008; Howe et al., 2017). In the present review, several studies (Kong et al., 2009, 2018; Gollub et al., 2018) were consistent that expectation can significantly enhance the analgesic effect of VA.

Interestingly, the oldest canonical classic of Chinese medicine, the *Yellow Emperor's Inner Classic* (*Huang Di Nei Jing*), has long recorded this. Written in the first century BCE, the text states that “if a patient does not consent to therapy with positive engagement, the physician should not proceed as the therapy will not succeed” (*SuWen* Chapter 11).

However, two studies (Tu et al., 2019; Yu et al., 2020) included in this review did not detect the expected significant differences between high and low context groups. Perhaps the method of creating expectation affected brain response and clinical efficacy, since gaining the trust of patients is a complicated process. Warmth and empathy maybe just two of several factors that can influence the expectations and beliefs of patients (Kong et al., 2009).

To date, research on the expectation effect has mainly involved analgesia, which found that although the expectation and acupuncture could achieve similar analgesic effects, their brain activation responses were different (Kong et al., 2009). Moreover, brain networks involved in expectation modulation can vary with the methods used to create expectations. Therefore, it may be important to investigate the most effective method to generate expectations in patients based on the standard expectation scale and with large sample size.

#### Different Acupoints

The choice of acupoints is a key factor affecting the therapeutic effect of acupuncture (Armour and Smith, 2016). It is even more important to understand the specific brain activation of each acupoint, but this is not straightforward. One previous study (Cho et al., 1998) has shown that acupuncture at BL67 activated the visual cortex, but the results have not been replicated (Siedentopf et al., 2002; Li et al., 2003). In addition, GB37 (Gareus et al., 2002) and GB43 (Wesolowski et al., 2009) were used to explore visual and auditory cortical activation, respectively, but the evidence is of insufficient quality.

All the three studies included in this review showed that multiple acupoints activated more brain regions and showed better efficacy than a single acupoint. Although the difference in efficacy was not statistically significant, the results indicated that the combined acupoints created broader stimulation of brain areas (Zhang et al., 2019), rather than a simple sum of the effects at more than one acupoint. However, some researchers found no difference between two-point and single-point stimulation (Alizadeh et al., 2014; Xing, 2016; Qu et al., 2020).

While there is broad agreement on acupoint number as a factor in the efficacy of acupuncture, some inconsistencies remain. Further comparison of brain responses between different single and multiple acupoints is imperative to establish the link between acupoints and diseases.

#### Location of Acupoints

According to TCM theory, diseases are caused by an imbalance between Yin and Yang. Therefore, balancing Yin and Yang is a key to treating diseases (Shuang et al., 2020). ON, as a method of achieving this balance, is widely used in the treatment of various diseases, including using the left acupoint to treat the right, using the lower acupoint to treat the upper, and using the front acupoint to treat conditions of the back, which produced beneficial effects. In this review, two studies (Zhang et al., 2018; Yan et al., 2020) used fMRI to explore the mechanism and efficacy of acupuncture at non-painful side ST38 on the painful side of chronic shoulder pain and between local and distal points. However, there is no clear definition in the literature on the distance that should be considered local or distant (Wong et al., 2015). The differences in efficacy and mechanism found between the two do provide evidence that can be used in the clinic, but long-term efficacy and prognosis still need to be further verified.

In summary, it can be seen from the above discussion that further research is needed to explore the mechanisms by which a range of factors influence the acupuncture effect. This is because the factors are numerous, and they are not limited to needle depth, *deqi*, disease stage, treatment course, and some non-specific effects, such as expectation effect and understanding of patients about acupuncture. Additional factors such as acupuncture time, manipulation, and duration need to be further investigated in the future.

## Limitations and Perspectives for Future Studies

Overall, acupuncture does have some effects on brain activation. The application of modern technology such as MRI, the diversification of analytical methods, and the development of algorithms make the study of brain responses more accurate. However, some limitations should be acknowledged. First, many non-acupuncture factors interfere with brain response to stimulation at acupoints. The form of the placebo, the psychological attitude of the participants toward acupuncture, and the illness of the patients were the three most interesting factors. Second, although this review has explored the effect of acupuncture and the factors influencing acupuncture efficacy from many perspectives, it cannot draw a reliable conclusion due to the small samples and large heterogeneity of the included studies. Third, while many studies have been conducted on the immediate effects of acupuncture, those evaluating brain responses to long periods of clinical acupuncture treatment are still lacking. Fourth, we did not include non-English studies; therefore, a language bias may be present.

In summary, there is much room for further research investigating the links among acupoint, brain activation, and disease type. First, the selection of appropriate SA is the basis of clinical effectiveness since SA type affects the assessment of the efficacy of acupuncture. Second, brain response differs between patients and HC. The response to acupuncture in pathological conditions is mainly concentrated at the pathology-related brain regions, so the same acupuncture in HC could not elicit a similar brain effect to stimulation at this acupoint. Third, standardization of MRI parameters and data collection may increase the homogeneity of results, and results such as coordinates should be made public to pave the way for verification studies and other future applications. Fourth, adherence to the Standards for Reporting Interventions in Clinical Trials of Acupuncture guidelines is necessary since the standardization of acupuncture methodology is very important to obtain reliable and precise results.

In brief, exploring the mechanism of acupuncture with imaging tools is a promising avenue for TCM. However, future developments, such as the accuracy of patient-specific predictions made by machine learning approaches, should be based on high-quality data, including the standardization of acupuncture and MRI parameters.

## Conclusions

In conclusion, MRI, as an advanced visualization method, facilitates a better understanding of the neural mechanisms of acupuncture. We found that stroke and GB34 were the most studied disease and acupoint, and rs-fMRI and FC were the most applied experimental and analytic methods. We found that the type of SA affected the efficacy of acupuncture and the brain response. Processing in the brain after acupuncture differs between patients and healthy individuals. The brain response to acupuncture in patients occurs mainly in disorder-related areas. The factors influencing the efficacy of acupuncture, including depth of needling, number and location of acupoints, *deqi*, and expectation effect, could be objectively assessed through brain responses. However, due to small sample sizes, different study designs, and analytical methods, the results were heterogeneous. Further studies with larger sample sizes, careful experimental design, and multimodal neuroimaging techniques, and standardized acupuncture and MRI methods should be conducted to better explain the efficacy and specificity of acupuncture, and to prepare for accurate efficacy prediction in the future.

## Author Contributions

JZ and JX designed the whole study, analyzed the data, and wrote the manuscript. ZhL and ZiL searched and selected the studies. JL participated in the interpretation of data. HY and QH offered good suggestions. All authors read and approved the final manuscript.

## Conflict of Interest

The authors declare that the research was conducted in the absence of any commercial or financial relationships that could be construed as a potential conflict of interest.

## Publisher's Note

All claims expressed in this article are solely those of the authors and do not necessarily represent those of their affiliated organizations, or those of the publisher, the editors and the reviewers. Any product that may be evaluated in this article, or claim that may be made by its manufacturer, is not guaranteed or endorsed by the publisher.
