# Supplementary material for: Progress of Acupuncture Therapy in Diseases Based on Magnetic Resonance Image Studies: A Literature Review
Source: Front Hum Neurosci. 2021 Aug 20;15:694919. doi: 10.3389/fnhum.2021.694919 (PMC8417610; doi:10.3389/fnhum.2021.694919)
Supplement: Supplementary file 1 [file Data_Sheet_1.doc]

**Supplementary Table 1. 107 studies of acupuncture for diseases based on MRI**

| Numbers | Author(year) | Subjects | MRI (1.5T/3T) | Intervention | Groups | **Were HC intervened (Y/N)** | Acupoints | Data analysis | Experimental design |
| --- | --- | --- | --- | --- | --- | --- | --- | --- | --- |
| 1 | Li et al. 2006[1] | 12 stroke/  12 HC | 1.5T | EA | VA/SA | Y | LI4 and LI11 | SPM | block/R(45s)-S(45s), 3 times |
| 2 | Napadow et al. 2007[2] | 13 Carpal Tunnel Syndrome/  12 HC | 3.0 T | MA | VA/SA | Y | LI4, SJ5, PC7 | FC | RS/ 3 times per week for three weeks and 2 times per week for the remaining two weeks. |
| 3 | Schaechter et al. 2007[3] | 7 stroke | 3T | MA | 4 VA/3 SA | / | N | GLM | RS/ twice weekly for 10 weeks. |
| 4 | Zhou et al. 2008[4] | 26 Alzheimer' s Disease | 1.5T | EA | / | / | left HT7, ST40, KI3 and ST36 | SPM | block/S(60s)-R(60s), 6 times |
| 5 | Wu et al. 2008[5] | 11 Spastic Cerebral Palsy/10 HC | 2T | MA | / | Y | left LI3 | SPM | block/S(1min)-R(1min),3 times |
| 6 | Zhang et al. 2009[6] | 12 left lower extremity pain | 3T | EA | / | NA | left GB34 and GB39 | SPM | 30 min  block/R(1min)-S(1min)-R(1min)-S(1min)-R(1min) |
| 7 | Chae et al. 2009[7] | 10 Parkinson’s Diseases/10 HC | 3T | MA | VA /  covert placebo/  overt placebo | N | left GB34 | SPM | block/R(2)-S(1min), 3 times |
| 8 | Li et al. 2010[8] | 7 aphasia stroke/14 HC | 1.5T | EA | / | Y | SJ 8 | SPM | block/R(45s)-S(45s), 3 times |
| 9 | Huang et al. 2011[9] | 12 ischemic stroke | 3T | MA | 6 VA/6 SA | NA | SJ5 | ReHo | block/S(30s)-R(30s), 6 times |
| 10 | Feng et al. 2012[10] | 12 MCI /12 HC | 3T | MA | DA/ SA | Y | KI3 | FC | NRER/R(1min)-S(DA/SA)(2min)-R(6min) |
| 11 | Liu et al. 2012[11] | 41 myopia | 1.5T | MA | 11 VA and TI/  11 VA and NI/  10 SA and TI/  9 SA and NI | / | LR3 | GLM | RS/R(186s)-S(180s) |
| 12 | Chu et al.2012[12] | 30 irritable bowel syndrome | 3T | EA | 15 EA vs 15 SA | / | bilateral ST36, ST37 and SP6 | SPM | block/30 min |
| 13 | Yeo et al.2012 | 12 Parkinson’s Disease/12 HC | 3T | MA | VA /SA | Y | right GB34 | ReHo | R(4min)-sham(S(1min)-R(1min-S(1min)-R(1min))-R(4min)-T1(10min)-verm(S(1min)-R(1min)-S(1min)-R(1min)-R(4min) |
| 14 | Ye et al.2012[14] | 20 low-back and leg pain patients | 1.5T | MA | 10 balanced acupuncture/  10 body acupuncture | / | balanced acupuncture：the Lumbago acupoint localized at the central point of the forehead  body acupuncture: BL40, BL25, Ashi acupoint and BL26 | FC, bilateral amygdala as the seed region | RS/20 min, twisted once every 5-10 minutes, using mild reinforcing-reducing method. |
| 15 | Wang et al.2012[15] | 8 MCI and 14 Alzheimer's Disease/  14 HC | 3T | MA | / | N | bilateral LR3 and LI4 | ReHo | block/R(min)-S(3min)-R(10min) |
| 16 | Huang et al.2013[16] | 10 ischaemic stroke | 3T | MA | / | / | SJ5 | SPM | tactile control (6min)-R(5min)-block/S(30s)-R(30s), 6 times |
| 17 | Chen et al.2013[17] | 10 ischemic stroke/6 HC | 3T | MA | / | Y | SJ5 | FC | block/R(30s)-S(30s), 6 times |
| 18 | Cho et al.2013[18] | 11 stroke/  10 HC | 3T | MA | / | Y | LI11 and ST36 | SPM | block/R(30s)-S(30s), 3 times |
| 19 | Maeda et al.2013[19] | 59 carpal tunnel syndrome | 3T | EA | 22 local VA/  18 distal VA/  19 SA | / | loca VA: PC7, SJ5;  distal acupoints (contralateral ankle, SP-6, LV-4,  SA: noninsertive needles placed over sham points, SH1 and SH2. | GLM | event-related/2-second stimulation events with randomized interstimulus interval (ISI), 6–12 seconds, and total scan time 5 minutes and 6 seconds, |
| 20 | Chen et al.2013[20] | 30 hypertension | 3T | MA | 15acupuncture/  15 no treatment | / | bilateral (except single points like DU20, DU23, and four points EXHN1, including ST9 , LI11,PC6, LI4, ST36, SP6, and LR3 | FC | RS/5 times 30 min acupuncture session for 5 days |
| 21 | Xie et al.2013[21] | 30 knee osteoarthritis | 3T | Mox | before vs after | / | Left ST 35 | fALFF and ReHo | RS/circling moxibustion for 30 s, bird-pecking moxibustion for 30 min, back and forth moving moxibustion for 30 min. |
| 22 | Kang et al.2013[22] | 25 smoke | 3T | MA | 12 VA/13 SA | / | left HT7 | SPM | block/S(60s)-R(60s), 3 times |
| 23 | Li et al.2013[23] | 63 Bell’s palsy/  39 HC | 1.5 T | MA | / | N | LI4 on the contralateral side of the facial palsy | GLM | Block10min(R(2min-S(2)-R(3)-S(2)-R(1)) |
| 24 | Zhou et al.2013[24] | 24 functional diarrhea/  24 HC | 3-T | MA | / | Y | ST25 | ReHo and FC, left ACC as the seed | RS/Both FD patients and HC accepted 10 sessions total of acupuncture treatment over a period of 2 weeks (5 sessions per week) |
| 25 | Quah-Smith et al.2013[25] | 10 depression/  10 HC | 3T | Laser Acupuncture | / | Y | LR14, LR8, RN14, and HT7 | ICA | task /block(2s delay-R(2s)-s(20s))/8times/session |
| 26 | Chen et al.2013[26] | 12 mild cognitive impairment/ 12 HC | 3T | MA | DA/SA | N | right KI3 | GLM | task /block/R(6min)-s(3min)-R(6min) |
| 27 | Bai et al.2013[27] | 12 mild cognitive impairment/ 12 HC | 3T | MA | / | N | right KI3 | Small-World Analysis | NRER/R(1min)-S(2min)-R(6min) |
| 28 | Li et al.2014[28] | 20 chronic low Back Pain1/10 HC | 3T | MA | / | N | bilateral BL23, lower back ah shi point, DU3, bilateral BL40 and KI3 | ICA | RS/30 min acupuncture treatments three times a week for 4 weeks (12 treatments in total). |
| 29 | Zhang et al.2014[29] | 8 ischemic stroke/10 HC | 3T | MA | / | Y | GB34 | SPM | NRER/R(1min)-S(1min)-R(8min) |
| 30 | Bai et al.2014[30] | 9 first-ever stroke/8 HC | 3T | MA | / | N | GB34 | GLM and TBSS | NRER/R(1min)-S(1min)-R(8min) |
| 31 | Xie et al.2014[31] | 9 stroke/8 HC | 3T | MA | / | Y | GB34 | GLM and GCA | NRER/R(1min)-S(1min)-R(8min) |
| 32 | Qi et al.2014[32] | 16 ischemic stroke | 3T | MA | 8 VA/8 SA | / | SJ5 | SPM | block /R(30s)-S(30s), total 6min6s |
| 33 | He et al.2014[33] | 28 Bell’s palsy/20 HC | 1.5T | MA | 18 early group/  21 late group/  19 recovered group | Y | LI4 on the contralateral side of paresis | FC, primary somatosensory area (SI) as the seed region | RS/10 min acupuncture , three times a week |
| 34 | Wang et al.2014[34] | 14 Alzheimer's Disease/  14 HC | 3T | MA | / | Y | bilateral LR3 and LI4 | FC, bilateral hippocampus as the seed region | block/R(3min)-S(3min)-R(10min) |
| 35 | Liang et al.2014[35] | 9 Alzheimer's Disease/  11 HC | 3T | MA | / | Y | right Liv3 and LI4 | ICA | block/R(3min)-S(3min)-R(10min) |
| 36 | Zhu et al.2014[36] | 28 irritable bowel syndrome | 1.5T | Mox | 15 VA/13 SA | / | ST25, RN6, and RN12 | ICA | RS/three times per week for four weeks |
| 37 | Li et al.2014[37] | 24 functional dyspepsia/24HC | 3T | MA | / | Y | right ST36 | GLM | block/R(1min)-S(1min)-R(1min)-S(1min)-R(1min)-S(1min)-R(1min) |
| 38 | Zhao et al.2014[38] | 40 migraine | 3T | MA | 20 active point/  20 inactive point | / | active point: bilateral SJ5 ,GB20 , GB34 , and GB40   inactive point: bilateral SJ22 , PC7 , GB37, and SP3 | ReHo | RS/4 times a week for 30 minutes each for 8 weeks |
| 39 | Hashmi et al.2014[39] | 40 knee osteoarthritis | 3 T | EA | 20VA/20SA | / | LI3 and LI4 | Graph-theoretic metrics of network topology | RS/the procedure (real or sham acupuncture) lasted 25 min. |
| 40 | Napadow et al.2014[40] | 14 atopic dermatitis | 3 T | EA | 1) verum, or real, electroacupuncture  2) placebo acupuncture  3) verum antihistamine solution a  4) placebo solution . | / | LI-11 and HT-3 | GLM | Block/a cross-over design |
| 41 | Yeo et al.2014[41] | 12 Parkinson's disease/12 HC | 3.0 T | MA | VA/SA | Y | GB34 | SPM | Block/SA:R(60s)-S(60s)-R(60s)-S(60s)-R(60s)-structural images(15min); then RA:R(60s)-S(60s)-R(60s)-S(60s)-R(60s) |
| 42 | Chen et al.2014a[42] | 6 ischemic stroke | 3T | MA | VA/SA | / | SJ5 | SPM | block/ rested for 5 min-sham acupuncture stimulus(6 min30s)-R(6min2s)-VA(6 min30s). |
| 43 | Chen et al.2014b[43] | 30 knee osteoarthritis | 3T | MA | 10 high dose VA/ 10 low dose VA/  10 sham group | / | high dose: GB34, SP9, GB39 and SP6, ST35 and Xi yan (extra point); low dose, ST35 and Xi yan (extra point), | FC, left posterior medial prefrontal cortex (pMPFC) , Cortical thickness analysis | RS/6 acupuncture treatment sessions in one month (twice per week for the first two weeks, once per week for the last two weeks |
| 44 | Shi et al.2015[44] | 24 acute low back pain | 3T | MA | / | / | BL40 | ReHo | block/R(30s)-S(30s), 6 times |
| 45 | Li et al.2015a[45] | 14 first-ever stroke patients | 3T | MA | 7 MA plus conventional drug/  7 conventional treatment group | / | GV20, GB20, bilateral GB39, LI11, LI4, ST36, and SP6 | DTI | RS/ 30 min, 5 days per week, four weeks. |
| 46 | Gao et al.2015[46] | 10 ischemic stroke/10 HC | 3T | MA | / | Y | ST36 | SPM | block/(30s)-S(30s), 6 times |
| 47 | Li et al.2015b[47] | 12 ischemic stroke | 3T | MA | / | / | SJ5 | SPM | block/(30s)-S(30s), 6times |
| 48 | Chen et al.2015[48] | 6 hemiplegic | 1.5T | MA | / | / | left GB34 | FC, right precentral gyrus as the seed | block/R(60s)-(S(30s)-R(30s),3times) |
| 49 | Fang et al.2015[49] | 8 functional dyspepsia/  10 HC | 1.5T | MA | / | N | ST36 and KI3,; additional acupoints included GB 41, PC 6, and HT 7. | FC, pregenual ACC, sub-ACC, MTL-hippocampus, IPL, and anterior insula | RS/20 min, three to four times a week for one month. |
| 50 | von Deneen et al.2015[50] | 19 overweight | 3T | MA | 10 MA/9 SA | / | bilateral ST 36 and SP 9 | BOLD | block/R(5min)-S(21min)-S(1min)-R(7 min) anatomical scan -(9 min) postscan |
| 51 | Wu et al.2015[51] | 20 Bell’s Palsy/20 HC | 1.5T | MA | / | Y | LI4, semi-individually at acupoints | FC, anterior cingulate gyrus (ACC) as the seed region | RS,Block/R(10min)-S(10min, Rotate needles10s every 2 min)-R(10min) |
| 52 | Egorova et al.2015[52] | 41 knee osteoarthritis | 3T | MA | VA at 6 acupoints/  VA at 2 acupoints/  SA | / | 2 acupoint: ST35 and Xi yan (extra point) and 6 acupoints: additionally at GB34, SP9, GB39 and SP6 | FC, periaqueductal gray (PAG) as the seed | RS/6 times 25 min acupuncture sessions in one month |
| 53 | Jia et al.2015[53][53] | 8 mild cognitive impairment/15 HC | 1.5T | MA | VA/SA | / | KI3 | ALFF | depth of about 20 mm and rotated right then left, at a frequency of 2 Hz for 60 s |
| 54 | Zhang et al.2016[54] | 12 migraine without aura/12 HC | 3T | MA | / | N | bilateral SJ23, GB8, GB20, EX-HN5, LI4, LR3, SJ5, GB34, and GB41. | FC | RS/30min, 5 times per week for 4 weeks |
| 55 | Li et al.2016[55] | 62 migraine/42 HC | 3T | MA | 11 VA1/  11 VA2 /  13 VA3 /  11 SA/ 16 waiting-list | N | VA1: GB34, GB40 and SJ5;  VA2: GB33, GB42 and SJ8;  VA3: ST36, ST42 and L16; SA: NAP1, NAP2 and NAP3 | FC, periaqueductal gray (PAG) as the seed region | RS/ 30 min, 5 times per week, four weeks. |
| 56 | Wang et al.2016[56] | 43 essential hypertension | 3T | MA | 15 LR3+KI3/  14 LR3/1  5 KI3 | / | LR3+KI3;LR3;KI3 | ReHo | RS/ 30 min, 5 times /week, two weeks. |
| 57 | Bao et al.2016[57][57] | 34 remissive Crohn’s disease/36 HC | 3T | EA, Mox | 16 EA/18 Mox | N | RN6, RN12, bilateral ST25 | ReHo | RS/30 minutes, three times/week for 12 week |
| 58 | Bian et al.2016[58] | 17 Bell’s palsy/20 HC | 1.5T | MA | / | N | left LI4 | FC, all the “peaks” of the activation areas as the seed region | block/RS(10min)-S( rotated for 10 seconds every 2 min, lasted 10 min)-R(10min) |
| 59 | Zheng et al.2016[59] | 28 hypertension | 3T | MA | 14 MA/14 SA | / | LR3 | FC, hypothalamus as the seed | RS/30 min, three times/week for 2 week |
| 60 | Wang et al.2016[60] | 46 depression | 3T | MA | 22 MA plus fluoxetine/  24 SA plus fluoxetine | / | RN12, RN10, RN6, RN4, KL 17, ST24, and Qipang. | FC, bilateral amygdala as the seed region | RS/once a day for the first three days and subsequently once every three days for the remainder of the 8-week trial. |
| 61 | Deng et al.2016[61] | 29 depression/29 HC | 3T | EA | / | N | DU20 | FC, the precuneus/posterior cingulate cortex (PC/PCC) as the seed | NRER/R(6min)-S(20min)-R(6min) |
| 62 | Li et al.2017a[62] | 17 subcortical stroke/14 HC | 3T | MA | 8 MA plus drug /  9 drug | N | DU20, GB20, bilateral GB-39, LI-11, LI-4, ST-36, SP-6 | FC, major cortical motor-related areas were selected using ROI-based correlation analysis | RS/ two hours a day for 5 days a week, one week a course, continuous four courses |
| 63 | Ning et al.2017[63] | 18 stroke/20 HC | 3T | MA | NA | N | GB34 | GLM, FC,   bilateral M1s as the seed region | NRER/R(1min)-S(1min)-R(8min) |
| 64 | Chang et al.2017[64] | 43 post-stroke motor aphasia | 3T | EA | 22 EA/21WT | / | HT 5,GB39 | GLM | block/R(30s)-S(30s), total 6min6s |
| 65 | Fu et al.2017[65] | 19 stroke / 17 HC | 3T | MA | / | N | GB34 | ICA | RS/R(8min 10s)-needing in (1min)-S(1min)-R(8min 10s),the structural imaging sans were conducted on each participant for 4 minutes and 10 seconds. |
| 66 | Li et al.2017b[66] | 62 migraine/  42 HC | 3T | MA | / | N | VA1: GB34, GB40 and SJ5.  VA2: GB33, GB42 and SJ8.  VA3: ST36, ST42 and L16.  SA: NAP1, NAP2 and NAP3 | ALFF | RS/30 min, 5 times/w, for 4 weeks |
| 67 | Wang et al.2017[67] | 36 major depressive disorder | 1.5T | MA | 18 abdominal acupuncture plus fluoxetine/  18 SA plus fluxetine | / | RN12, RN10, RN6, RN4), KL17, ST24, and Qipang. | seed-based correlation analysis | RS/once a day for the first three days and subsequently once every three days for the reminder of the 8-week trial.fluoxetine (20 mg) by mouth once per day |
| 68 | Liu et al.2017[68] | 184 migraine/  50 HC | 3T | MA | 98 MA/80 SA | N | bilaterally GB20, GB40, or GB41 or GB42, DU20, LR3, SJ 3 or SJ5, and extra point Taiyang | VBM and FC, left medial prefrontal cortex as the seed | RS/30 min, 3times/w for 8 weeks |
| 69 | Bao et al.2017[69] | 38 remissive Crohn’s disease | 3T | EA/Mox | 18 EA/20 Mox | / | bilateral ST25 , CV6 and CV12 . | seed-based correlation analysis | RS/30min, 3 days per week for a total of 12 weeks. |
| 70 | Tan et al.2017[69] | 32 mild cognitive impairment | 3T | MA | 16 MA/16 SA | / | EX-HN1, EX-HN3, PC6, KI3, ST40, and LR3 | FC, pregenual ACC, sub-ACC, MTL-hippocampus, IPL, and anterior insula as the seed region | RS/five times/week, for 4 consecutive weeks. |
| 71 | Makary et al.2018[71] | 47 low back pain | 3T | MA | 28 VA/19 PHNT | / | bilateral SP13, left SP11, and left ST36 | GLM | EREP/S(2s)-R( 7.9 ± 1.7s),5 times |
| 72 | Gollub et al.2018[72] | 43 knee osteoarthritis | 3T | MA | 21 MA/22 SA | / | right Liv3 and LI4 | ReHo | RS/five times a week, on weekdays, for 4 consecutive weeks. |
| 73 | Yeo et al.2018[73] | 10 Parkinson’s disease | 3T | EA | / | / | the right GB34 and the right LR3. | ReHo | RS/15min, 2 times per week, for 8 weeks |
| 74 | Li et al.2018[74] | 35 Parkinson’s disease | 3T | MA | 14 MA/  11 SA/  10 WT | / | DU20, bilateral  GB20 , and the Chorea-Tremor Controlled Zone | DC,ReHo,ALFF | RS/30 min, 2 times per week, for 12weeks |
| 75 | Pang et al.2018[75] | 23 premenstrual syndrome | 3T | EA | NA | / | SP6 | ReHo | NRER/R(6min)-S(6min)-R(6min) |
| 76 | Zhao et al.2018[76] | 60 constipation-predominant irritable bowel syndrome | 1.5T | EA/Mox | 30 EA/30 Mox | / | bilateral ST 25 and ST 37 | / | block/30 s of distension alternated with 30 s of  balloon inflation, 3 time |
| 77 | Zhang et al.2018[77] | 20 unilateral chronic shoulder pain | 3T | MA | 9 contra-acupuncture/  11ipsi-acupuncture | / | non-painful side ST38/painful side ST38 | ReHo | RS/contra- or ipsi-acupuncture treatment for 20 min |
| 78 | Kong et al.2018[78] | 46 knee osteoarthritis | 3T | MA | 17 boosted acupuncture/ 17 standard acupuncture/12 VA controls treatment | / | ST35, Xi yian (extrapoint), GB34, SP9, GB39 and SP6 | FC, nucleus accumbens (NAc) as the seed region | RS/20min, 2 times/week for the first two weeks, and 1 time/week for the last two weeks |
| 79 | Zeng et al.2018[79] | 3 Cancer-Related Cognitive Impairment/  15HC | 3T | MA | / | N | EX-HN1, EX-HN3, bilatera EX-HN5 , GB8 , GB15, GB20 , DU20, and ST8 | Fractional anisotropy (FA) | RS/30 min, 2 times/w, for 5 weeks |
| 80 | Zheng et al.2018[80] | 14 Alzheimer disease/  14 HC | 3T | MA | / | N | bilateral Liv3 and LI4 | ALFF and FC | block/R(3min)- S(3min)-R(10min) |
| 81 | Wu et al.2018[81] | 21 ischemic stroke | 3T | MA | 11 acupuncture plus conventional treatments/10 only conventional treatments | / | DU20, GB20, LI11, LI4, GB34, ST36, SP6, and GB39 | VBM | RS/30min, 2 times/week for 5 weeks |
| 82 | Lee et al.2019[82] | 43 low back pain | 3T | MA | 25VA/18 PHNT | / | bilateral SP13, left SP11, and left ST36 | ICA | RS/7 min acupuncture |
| 83 | Tu et al.2019[83] | 50 chronic low Back Pain | 3T | MA | 12 "augmented context” + VA/12 “limited context” + VA/13 “augmented context” + SA/13 “limited context” + SA | / | GV3, bilateral BL23, BL40, KI3, and 1–3 ashi points bilaterally on the lower back and legs | ICA | RS/ 25min, twice a week for 2 weeks and then once a week for 2 weeks |
| 84 | Xiang et al.2019[84] | 12 chronic low back pain | 3T | MA | / | / | the ankle zone 5 | ALFF | RS/Tactile stimulation(3s)-R(8min)-T1(15min)-S(3s)-R(8min) |
| 85 | Han et al.2019[85] | 22 poststroke motor impairment/22 HC | 3T | MA | / | Y | GB34 | TBSS and FC | NRER/R(8 min10sec)-S(1min)-R(8 min10sec) |
| 86 | Yu et al.2019[86] | 9 Parkinson’s disease | 3T | MA | 9 Parkinson’s disease/6 WT | / | DU20, Shen Guan, GB34 | FC | RS/Event-Related fMRI |
| 87 | Zou et al.2019[87] | 35 chronic migraine/  25 HC | 3T | MA | / | N | bilateral SJ5, GB20,GB8 and ST8 | ICA, SCA | RS/30 min, 3times/week for 12 weeks |
| 88 | Su et al.2019[88] | 16 primary dysmenorrheal | 1.5T | MA | / | / | bilateral Sanyinjiao and Diji | ALFF | RS/Rest 1 (R1), S(60s), and structural scan  and Rest (R2). |
| 89 | Zhang et al.2019[89] | 30 hypertension | 3T | MA | 10 LR3+KI3/  10 LR3/  10 KI3 | / | bilateral LR3、KI3/bilateral LR3/bilateral KI3 | ReHo | RS/1 min at intervals of 10 min during the 30 min treatment. |
| 90 | Wang et al.2019[90] | 20 tobacco dependence/20 HC | 1.5T | MA | / | N | LU7, LI4, DU20, ST36, SP6 and LR3 | fALFF | RS/30 min |
| 91 | Duan et al.2019[91] | 30 depression/29 HC | 3T | EA | / | N | DU20 | FC, amygdala as the seed region | NRER/20min |
| 92 | Yu et al.2020[86,92] | 50 chronic low Back Pain | 3T | MA | 12 "augmented context” +VA/  12 “limited context” +VA/  13 “augmented context” + SA/  13 “limited context” + SA | / | DU3, bilateral BL23, BL40, KI3, and 1-3 ashi points bilaterally on the lower back and legs | ICA | RS/25min, two times per week for the first two weeks and one time per week for the last two weeks. |
| 93 | Kim et al.2020[93] | 105 chronic low back pain/50 HC | 3T | MA/LA | 18 VA/18 SA/19 mock laser acupuncture /23 usual care | N | DU3, BL23 bilateral BL40 and KI-3 combined with 2-3 bilateral ah-shi | VBM,DTI | RS/20min, two treatments/week for 2 weeks; one treatment/week for 2 weeks |
| 94 | Chen et al.2020[94] | 10 ischemic stroke | 3T | MA | / | / | LI11 and ST36 | ReHo | RS/R(5min)-S(15min)-R(5min) |
| 95 | Han et al.2020[95] | 26 ischemic stroke/21HC | 3T | MA | / | Y | GB34 | Graph Theoretical Network Analysis | RS/R(8min10s)-S(60s)-R(8min10s) |
| 96 | Ren et al.2020[96] | 45 obesity | 3T | EA | acute effects:  26 VA/19 SA;  long-term effects:  17 VA/15 SA | / | RN12, RN10, ST25, SP15, ST21, ST24, ST26, ST27, SP14; additional distribution acupoints included ST37, ST39, LI11, ST44, TE6, LI4)and ST40  bilateral ST25, ST21, ST37, and ST44 | ALFF | RS/ acute effects:32min; long effect:three times a week for a course of 6–8 weeks |
| 97 | Liu et al.2020[97] | 12 nonacute sciatica/  15 HC | 3T | MA | / | N | six or 18 acupoints of manual acupuncture. The six acupoints were BL 23, GB 30, BL 40, GB 34, BL 60, and GB 39. The eighteen acupoints :BL 23, BL 25, BL 27, GB 30, BL 37, BL 54, BL 36, GB 31, BL 40, ST 36, GB 34, SP 9, BL 58, SP 6, GB 39, BL 60, KI 3, and BL 62. | ReHo; FC,PCC as the seed region | RS/20min, twice weekly for 4 weeks |
| 98 | Yan et al.2020[98] | 20 chronic shoulder pain | 3T | MA | 12 Contra-group-painful side/8 Ipsi-group( non-painful side (right) | / | non-painful side ST38/painful side ST38 | Voxel-Wise Degree Centrality Analysis | RS/20min |
| 99 | Wang et al.2020a[99] | 45 Primary insomnia | 3T | MA | 15 S-Acugroup/  15 M-Acu group/  15 N-Acu group | / | single acupoints: bilateral HT7 ;  multi-acupoints group: combination of bilateral HT-7, bilateral SP6 and single DU20;  Non-acupoint group: bilateral sham point at the junction point between the biceps brachii muscle and deltoid muscle | fALFF | RS/30 min, 5 times/w, for 5 weeks |
| 100 | Zhang et al.2020[100] | 29 essential hypertension | 3T | MA | 15 LR3＋K13/  14 LR3 and a sham location | / | LR3＋K13 | ALFF | RS/30 min, 5 times/w, for 2 weeks |
| 101 | Mawla et al.2020[101] | 76 Fibromyalgia Pain | 3T | EA | 38 EA/  38 Mock Laser acupuncture(ML) | / | right side LI11 to LI4, left side GB34 to SP6, and bilateral ST36. | seed-to-voxel correlation analysis | RS/25min, twice a week for four weeks |
| 102 | Shi et al.2020[102] | 30 primary insomnia | 3T | EA | / | / | HT7 | fALFF | RS/30min, five times a week for 5 weeks |
| 103 | Li et al.2020[103] | 45 migraine | 3T | MA | 12 VA1/13 VA2/14 VA3/13 SA/13 WT | / | VA1: GB34, GB40 and SJ5.  VA2: GB33, GB42 and SJ8.  VA3: ST36, ST42 and L16.  SA: NAP1, NAP2 and NAP3 | fALFF | RS/30min, five times a week for 4 weeks |
| 104 | Ma et al.2020[104] | 24 Diarrhea-Dominant Irritable Bowel Syndrome/25 HC | 3T | MA | / | N | DU20, EX-HN3, bilateral LR3, ST36, SP6, ST25, and ST37 | FC | RS/30min, three times a week for 6 weeks |
| 105 | Wang et al.2020b[105] | 30 internet addiction / 30 HC | 3T | MA | / | N | DU20, EX-HN1, and bilateral LI4, PC6, HT7, LR3, SP6, and GB39 | seed-to-voxel and ROI-to-ROI analyses | RS/30min, 5 times/week for 5 weeks |
| 106 | Pang et al.2020[106] | 23 premenstrual syndrome | 3T | EA | / | / | SP6 | FC, amygdala as the seed region | NRER/R(6s)-S(6s)-R(6s) |
| 107 | Sun et al.2020[106] | 70 functional dyspepsia | 3T | MA | 35 deqi/35 without deqi | / | RN12 and bilateral ST36 | FC, amygdala as the seed region | RS/30min, 5 times/week for 4 weeks |

Supplementary Table 2. Studies on brain response of influential factors

| Numbers | Author  (year) | Diseases | Intervention | design of study (T/C)(n) | Points | Data analysis | Experimental design/Treatment | Results |
| --- | --- | --- | --- | --- | --- | --- | --- | --- |
| **Depth** | | | | | | | | |
| 1 | Feng et al. 2012[10] | 12 mild cognitive impairment/  12 HC | MA | DA/ SA | KI3 | FC | NRER/R(1min)-S(DA/SA)  (2min)-R(6min) | Compared to SA, significantly increased correlations related with the temporal regions were found for the DA condition. These regions are implicated in memory encoding and retrieving. |
| 2 | Chen et al.2013[26] | 12 mild cognitive impairment/  12 HC | MA | DA/SA | right KI3 | GLM | task /block/R(6min)-s(3min)-R  (6min) | KI3 in MCI-Deep can induce the strongest and more extensive effective connectivity related to the therapeutic effect of acupuncture for the treatment of MCI. |
| 3 | Bai et al.2013[27] | 12 mild cognitive impairment/  12 HC | MA | DA/ SA | right KI3 | Small-World Analysis | NRER/R(1min)-S(2min)-R  (6min) | Deep acupuncture enhanced the compensatory of the loss in small world attributes for treatment effects. |
| ***Deqi*** | | | | | | | |  |
| 1 | Li et al.2015[45] | ischemic stroke | MA | 12 deqi /  non deqi patients | right SJ5 | SPM | block/S(30s)-R(30s), 6times | Compared with the non-Deqi group, the Deqi group exhibited marked activation of the right anterior lobe of the cerebellum and right limbic lobe (BA30). |
| 2 | Sun et al.2020[107] | FD | MA | 35 deqi FD patients /  35 without deqi FD patients | CV12 and bilateral ST36 | Seed-based functional connectivity | RS/30min, 5 times/week for 4 weeks | Compared with the non-Deqi group, the deqi group could significantly improve dyspepsia symptoms,which is related to influencing thebasolateral amygdala (BLA) and centromedial amygdala (CMA) rsFC |
| **Expectation** | | | | | | | | |
| 1 | Liu et al. 2012[11] | 41 myopia | MA | 11 VA and TI/  11 VA and NI/  10 SA and TI/  9 SA and NI | LR3 | GLM | RS, R(186s)-S(180s) | Activation of the cerebellum in this study was related to expectancy. |
| 2 | Hashmi et al.2014[39] | 40 knee osteoarthritis | EA | 20VA/20SA | LI3 and LI4 | Graph-theoretic metrics of network topology | RS, (real or sham acupuncture) lasted 25 min. | Psychological factors are able to induce experimental analgesia (as indicated by reduction in evoked pain) in chronic knee OA pain patients |
| 3 | Kong et al.2018[78] | 46 knee osteoarthritis | MA | 17 boosted acupuncture/ 17 standard acupuncture/  12 VA controls treatment | ST35, Xi yian (extrapoint), GB34, SP9, GB39 and SP6 | FC, nucleus accumbens (NAc) as the seed region | RS/20min, 2 times/week for the first two weeks, and 1 time/week for the last two weeks | FC increases between the NAc and the medial prefrontal cortex (MPFC)/rostral anterior cingulate cortex (rACC) and dorsolateral prefrontal cortex in the boosted group as compared to the standard acupuncture group after multiple treatments. |
| 4 | Gollub et al.2018[72] | 43 knee osteoarthritis | MA | 21 MA/22 SA | right Liv3 and LI4 | ReHo | RS, five times/week, for 4 consecutive weeks. | Expectancy significantly and similarly modulates the pain experience in knee OA patients in both verum and sham acupuncture, However, there were different patterns of changes in fMRI indices of brain activity associated with VA and SA modalities specifically in the lateral prefrontal cortex. |
| 5 | Tu et al.2019 | 50 chronic low Back Pain | MA | 12 "augmented context” + VA/12 “limited context” + VA/13 “augmented context” + SA/13 “limited context” + SA | GV3, bilateral BL23, BL40, KI3, and 1–3 ashi points bilaterally on the lower back and legs | ICA | RS/ 25min, twice a week for 2 weeks and then once a week for 2 weeks | No significant difference was observed between the augmented and limited context groups in pain severity； |
| **Number of acupoints** | | | | | | | | |
| 1 | Wang et al.2016[56] | 43 essential hypertension | MA | 15 LR3+KI3/  14 LR3/  15 KI3 | LR3+KI3;  LR3;KI3 | ReHo | RS, 30 min, 5 times /week, two weeks. | LR3+KI3 acupoints combination may generate a synergistic effect, and it is not simple sum of single acupoint effect. |
| 2 | Zhang et al.2019[89] | 30 hypertension | MA | 10 LR3+KI3/  10 LR3/  10 KI3 | bilateral LR3 and KI3/bilateral LR3/bilateral KI3 | ReHo | RS, 1 min at intervals of 10 min during the 30 min treatment. | Combined acupoints of LR3 and KI3 could act on wider brain areas than the sum of single acupoints, whose functions include emotional processing, cognition, somatic sensation, spatial orientation, language production, and vision. |
| 3 | Wang et al.2020a[99] | 45 Primary insomnia | MA | 15S-Acu/  15 M-Acu/  15 N-Acu | S-Acu: bilateral HT7;  M-Acu: bilateral HT-7 and SP6 and single DU20; N-Acu: bilateral sham point | fALFF | RS, 30 min, 5 times/w, for 5 weeks | The combination of multi-acupoints might lead to a better clinical efficacy and could impact more regions which were influenced by the sleep experience |
| **Location of acupoints** | | | | | | | | |
| 1 | Zhang et al.2018[77] | 20 unilateral chronic shoulder pain | MA | 9 contra-group/  11 ipsi-group | non-painful side ST38/painful side ST38 | ReHo | RS, contra- or ipsi-acupuncture treatment for 20 min | Clinical improvement in decreasing pain intensity and increasing shoulder function in both groups, and the mean objective shoulder functional improvement in contra group was better than that in ipsi-group. The brain mechanism of contra-acupuncture at ST 38 was distinguishable from ipsi-acupuncture regarding ReHo values |
| 2 | Yan et al.2020[98] | 20 chronic shoulder pain | MA | 12 contra-group/  8 ipsi-group | non-painful side ST38/painful side ST38 | Voxel-Wise Degree Centrality Analysis | RS, 20min | Increased DC in the anterior/paracingulate cortex and decreased DC in bilateral postcentral gyriwere found in the contra-group, while decreased DC in the bilateral cerebellum and right thalamus was observed in the ipsi-group. the DC value in the bilateral anterior/paracingulate cortex was positively correlated with the treatment-related change in the Constant–Murley score. |
| 3 | Maeda et al.2013[19] | 59 carpal tunnel syndrome | EA | 22 local VA/18 distal VA/19 SA | loca VA: PC7, SJ5; distal VA, SP-6, LV-4, SA: sham points, | GLM | RS, event-related/2-second stimulation events with randomized interstimulus interval (ISI), 6–12 seconds, and total scan time 5 minutes and 6 seconds, | VAS scores for paresthesia showed significant reductions for the local group but not for the distal. In terms of brain response, consistent activation was found in bilateral insulae, secondary somatosensory cortex (S2), local EA produced activation in bilateral superior temporal gyrus (STG) and contralateral postcentral gyrus (S1), with deactivation in cuneus and ipsilateral S1, no deactivation and other activation were found in distal EA. |

Abbreviations: ACC, anterior cingulate cortex; DC, degree centrality; EA, Electroacupuncture; fALFF, fractional amplitude of low-frequency fluctuations; FC, functional connectivity analysis; GLM, general linear model; HC, health controls; ICA, independent component analysis; MA, manual acupuncture; M: multiple; /: irrelevant ; NI, nontreatment instruction; N, no; NRER, non-repeated event-related; RS, resting state; ReHo, regional homogeneity; SA: sham acupuncture; S, single; TI: treatment instruction; VA, verum acupuncture; Y, yes; Acupoints: BL23, Shenshu; BL25, Dachangshu; BL40, Weizhong; BL62, Shenmai; BL26, Guanyuanshu; BL36, Chengfu; BL54, Zhibian; BL60, Kunlun; BL58, Feiyang; BL27, Xiaochangshu; BL37, Yinmen; DU3, Yaoyangguan; DU20, Baihui; DU23, Shangxing; DU29, Yintang; EX-HN5,Taiyang; EX-HN1, Sishenchong; GB8, Shuaigu; GB30, Huantiao; GB31, Fengshi; GB33,Xiyangguan; GB39, Xuanzhong; GB40, Qiuxu; GB41, Zulinqi; HT3, Shaohai; HT5, Tongli; HT7, Shenmen; KI3, Taixi; LI3, Sanjian; LI4, Hegu; LI6, Pianli; LI7, Lieque; LR8, Ququan; LR14,Jimen; LR3, Taichong; PC6, Neiguan; PC7, Daling; PC8, Laogong; RN4, Guanyuan; RN6,Qihai; RN10, Xiawan; RN12, Zhongwan;RN14, Juque; SJ6, Zhigou; SJ5, Waiguan; SJ8, Sanyangluo; SJ22, Erheliao; SJ23, Sizhukong; SP6, Sanyinjiao; SP8, Diji; SP9, Yinlingquan; SP3, Taibai; SP14, Fujie; SP11, Jimen; SP13, Fushe; SP15, Daheng; ST6, Jiache; ST9, Renying; ST21,Liangmen; ST24, Huaroumen; ST25, Tianshu; ST26, Wailing; ST27, Daju; ST35, Dubi; ST36 , Zusanli; ST37, Shangjuxu; ST38, Tiaokou; ST39, Xiajuxu; ST40, Fenglong; ST42, Chongyang; ST44, Neiting.

**References:**

1. Li G, Jack CR, Yang ES. An fMRI study of somatosensory-implicated acupuncture points in stable somatosensory stroke patients. J MAGN RESON IMAGING 2006;24:1018-1024

2. Napadow V, Kettner N, Liu J, et al. Hypothalamus and amygdala response to acupuncture stimuli in Carpal Tunnel Syndrome. PAIN 2007;130:254-266

3. Schaechter JD, Connell BD, Stason WB, et al. Correlated Change in Upper Limb Function and Motor Cortex Activation After Verum and Sham Acupuncture in Patients with Chronic Stroke. The Journal of Alternative and Complementary Medicine 2007;13:527-532

4. Zhou Y, Jin J. Effect of acupuncture given at the HT 7, ST 36, ST 40 and KI 3 acupoints on various parts of the brains of Alzheimer' s disease patients. ACUPUNCTURE ELECTRO 2008;33:9

5. Wu Y, Jin Z, Li K, et al. Effect of Acupuncture on the Brain in Children With Spastic Cerebral Palsy Using Functional Neuroimaging (fMRI). J CHILD NEUROL 2008;23:1267-1274

6. Zhang JH, Li J, Cao XD, Feng XY. Can electroacupuncture affect the sympathetic activity, estimated by skin temperature measurement? A functional MRI study on the effect of needling at GB 34 and GB 39 on patients with pain in the lower extremity. Acupunct Electrother Res 2009;34:151-164

7. Chae Y, Lee H, Kim H, et al. Parsing brain activity associated with acupuncture treatment in Parkinson's diseases. MOVEMENT DISORD 2009;24:1794-1802

8. Li G, Yang ES. An fMRI study of acupuncture-induced brain activation of aphasia stroke patients. COMPLEMENT THER MED 2011;19:S49-S59

9. Huang Y, Xiao H, Chen J, et al. Needling at the Waiguan (SJ5) in healthy limbs deactivated functional brain areas in ischemic stroke patients A functional magnetic resonance imaging study. NEURAL REGEN RES 2011;6:2829-2833

10. Feng Y, Bai L, Ren Y, et al. FMRI connectivity analysis of acupuncture effects on the whole brain network in mild cognitive impairment patients. MAGN RESON IMAGING 2012;30:672-682

11. Liu H, Xu J, Shan B, et al. Determining the precise cerebral response to acupuncture: an improved FMRI study. PLOS ONE 2012;7:e49154

12. Chu WC, Wu JC, Yew DT, et al. Does Acupuncture Therapy Alter Activation of Neural Pathway for Pain Perception in Irritable Bowel Syndrome?: A Comparative Study of True and Sham Acupuncture Using Functional Magnetic Resonance Imaging. J NEUROGASTROENTEROL 2012;18:305-316

13. Yeo S, Lim S, Choe IH, et al. Acupuncture stimulation on GB34 activates neural responses associated with Parkinson's disease. CNS NEUROSCI THER 2012;18:781-790

14. Ye Y, Liu B. Analgesic effects of balanced acupuncture versus body acupuncture in low-back and leg pain patients with lumbar disc herniation, as assessed by resting-state functional magnetic resonance imaging. NEURAL REGEN RES 2012;7:1624-1629

15. Wang Z, Nie B, Li D, et al. Effect of Acupuncture in Mild Cognitive Impairment and Alzheimer Disease: A Functional MRI Study. PLOS ONE 2012;7:e42730

16. Huang Y, Chen J, Lai X, et al. Lateralisation of Cerebral Response to Active Acupuncture in Patients with Unilateral Ischaemic Stroke: An Fmri Study. ACUPUNCT MED 2013;31:290-296

17. Chen J, Huang Y, Lai X, et al. Acupuncture at Waiguan (TE5) influences activation/deactivation of functional brain areas in ischemic stroke patients and healthy people: A functional MRI study. NEURAL REGEN RES 2013;8:226-232

18. Cho SY, Kim M, Sun JJ, et al. A comparison of brain activity between healthy subjects and stroke patients on fMRI by acupuncture stimulation. CHIN J INTEGR MED 2013;19:269-276

19. Maeda Y, Kettner N, Lee J, et al. Acupuncture-Evoked Response in Somatosensory and Prefrontal Cortices Predicts Immediate Pain Reduction in Carpal Tunnel Syndrome. EVID-BASED COMPL ALT 2013;2013:1-13

20. Chen H, Dai J, Zhang X, et al. Hypothalamus-Related Resting Brain Network Underlying Short-Term Acupuncture Treatment in Primary Hypertension. EVID-BASED COMPL ALT 2013;2013:1-9

21. Xie H, Xu F, Chen R, et al. Image formation of brain function in patients suffering from knee osteoarthritis treated with moxibustion. J TRADIT CHIN MED 2013;33:181-186

22. Kang OS, Kim SY, Jahng GH, et al. Neural substrates of acupuncture in the modulation of cravings induced by smoking-related visual cues: an fMRI study. Psychopharmacology (Berl) 2013;228:119-127

23. Li C, Yang J, Sun J, et al. Brain Responses to Acupuncture Are Probably Dependent on the Brain Functional Status. EVID-BASED COMPL ALT 2013;2013:1-14

24. Zhou S, Zeng F, Liu J, et al. Influence of Acupuncture Stimulation on Cerebral Network in Functional Diarrhea. EVID-BASED COMPL ALT 2013;2013:1-9

25. Quah-Smith I, Suo C, Williams MA, Sachdev PS. The Antidepressant Effect of Laser Acupuncture: A Comparison of the Resting Brain's Default Mode Network in Healthy and Depressed Subjects During Functional Magnetic Resonance Imaging. Medical Acupuncture 2013;25:124-133

26. Chen S, Bai L, Xu M, et al. Multivariate Granger Causality Analysis of Acupuncture Effects in Mild Cognitive Impairment Patients: An fMRI Study. EVID-BASED COMPL ALT 2013;2013:1-12

27. Bai L, Zhang M, Chen S, et al. Characterizing AcupunctureDe Qi in Mild Cognitive Impairment: Relations with Small-World Efficiency of Functional Brain Networks. EVID-BASED COMPL ALT 2013;2013:1-8

28. Li J, Zhang JH, Yi T, et al. Acupuncture treatment of chronic low back pain reverses an abnormal brain default mode network in correlation with clinical pain relief. ACUPUNCT MED 2014;32:102-108

29. Zhang Y, Li K, Ren Y, et al. Acupuncture Modulates the Functional Connectivity of the Default Mode Network in Stroke Patients. EVID-BASED COMPL ALT 2014;2014:1-7

30. Bai L, Tao Y, Wang D, et al. Acupuncture Induces Time-Dependent Remodelling Brain Network on the Stable Somatosensory First-Ever Stroke Patients: Combining Diffusion Tensor and Functional MR Imaging. Evid Based Complement Alternat Med 2014;2014:740480

31. Xie Z, Cui F, Zou Y, Bai L. Acupuncture Enhances Effective Connectivity between Cerebellum and Primary Sensorimotor Cortex in Patients with Stable Recovery Stroke. EVID-BASED COMPL ALT 2014;2014:1-9

32. Qi J, Chen J, Huang Y, et al. Acupuncture at Waiguan (SJ5) and sham points influences activation of functional brain areas of ischemic stroke patients: a functional magnetic resonance imaging study. NEURAL REGEN RES 2014;9:293-300

33. He X, Zhu Y, Li C, et al. Acupuncture-induced changes in functional connectivity of the primary somatosensory cortex varied with pathological stages of Bell’s palsy. NEUROREPORT 2014;25:1162-1168

34. Wang Z, Liang P, Zhao Z, et al. Acupuncture modulates resting state hippocampal functional connectivity in Alzheimer disease. PLOS ONE 2014;9:e91160

35. Liang P, Wang Z, Qian T, Li K. Acupuncture Stimulation of Taichong (Liv3) and Hegu (LI4) Modulates the Default Mode Network Activity in Alzheimer’s Disease. American Journal of Alzheimer's Disease & Other Dementiasr 2014;29:739-748

36. Zhu Y, Wu Z, Ma X, et al. Brain regions involved in moxibustion-induced analgesia in irritable bowel syndrome with diarrhea: a functional magnetic resonance imaging study. BMC Complement Altern Med 2014;14:500

37. Li Z, Zeng F, Yang Y, et al. Different Cerebral Responses to Puncturing at ST36 among Patients with Functional Dyspepsia and Healthy Subjects. Forschende Komplementärmedizin / Research in Complementary Medicine 2014;21:1

38. Zhao L, Liu J, Zhang F, et al. Effects of long-term acupuncture treatment on resting-state brain activity in migraine patients: a randomized controlled trial on active acupoints and inactive acupoints. PLOS ONE 2014;9:e99538

39. Hashmi JA, Kong J, Spaeth R, et al. Functional Network Architecture Predicts Psychologically Mediated Analgesia Related to Treatment in Chronic Knee Pain Patients. J NEUROSCI 2014;34:3924-3936

40. Napadow V, Li A, Loggia ML, et al. The Brain Circuitry Mediating Antipruritic Effects of Acupuncture. CEREB CORTEX 2014;24:873-882

41. Yeo S, Choe IH, van den Noort M, et al. Acupuncture on GB34 activates the precentral gyrus and prefrontal cortex in Parkinson's disease. BMC Complement Altern Med 2014;14:336

42. Chen J, Wang J, Huang Y, et al. Modulatory effect of acupuncture at Waiguan (TE5) on the functional connectivity of the central nervous system of patients with ischemic stroke in the left basal ganglia. PLOS ONE 2014;9:e96777

43. Chen X, Spaeth RB, Retzepi K, Ott D, Kong J. Acupuncture modulates cortical thickness and functional connectivity in knee osteoarthritis patients. SCI REP-UK 2015;4

44. Shi Y, Liu Z, Zhang S, et al. Brain Network Response to Acupuncture Stimuli in Experimental Acute Low Back Pain: An fMRI Study. EVID-BASED COMPL ALT 2015;2015:1-13

45. Li Y, Wang Y, Zhang H, Wu P, Huang W. The Effect of Acupuncture on the Motor Function and White Matter Microstructure in Ischemic Stroke Patients. EVID-BASED COMPL ALT 2015;2015:1-10

46. Gao Y, Lin Z, Tao J, et al. Evidence of timing effects on acupuncture: A functional magnetic resonance imaging study. EXP THER MED 2015;9:59-64

47. Li MK, Li YJ, Zhang GF, et al. Acupuncture for ischemic stroke: cerebellar activation may be a central mechanism following Deqi. NEURAL REGEN RES 2015;10:1997-2003

48. Chen X, Zhang H, Zou Y. A functional magnetic resonance imaging study on the effect of acupuncture at GB34 (Yanglingquan) on motor-related network in hemiplegic patients. BRAIN RES 2015;1601:64-72

49. Fang J, Wang D, Zhao Q, et al. Brain-Gut Axis Modulation of Acupuncture in Functional Dyspepsia: A Preliminary Resting-State fcMRI Study. EVID-BASED COMPL ALT 2015;2015:1-11

50. von Deneen KM, Qin W, Liu P, et al. Connectivity Study of the Neuromechanism of Acute Acupuncture Needling during fMRI in “Overweight” Subjects. EVID-BASED COMPL ALT 2015;2015:1-12

51. Wu H, Kan H, Li C, et al. Effect of Acupuncture on Functional Connectivity of Anterior Cingulate Cortex for Bell’s Palsy Patients with Different Clinical Duration. EVID-BASED COMPL ALT 2015;2015:1-7

52. Egorova N, Gollub RL, Kong J. Repeated verum but not placebo acupuncture normalizes connectivity in brain regions dysregulated in chronic pain. NeuroImage: Clinical 2015;9:430-435

53. Jia B, Liu Z, Min B, et al. The Effects of Acupuncture at Real or Sham Acupoints on the Intrinsic Brain Activity in Mild Cognitive Impairment Patients. EVID-BASED COMPL ALT 2015;2015:1-9

54. Zhang Y, Li KS, Liu HW, et al. Acupuncture treatment modulates the resting-state functional connectivity of brain regions in migraine patients without aura. CHIN J INTEGR MED 2016;22:293-301

55. Li Z, Liu M, Lan L, et al. Altered periaqueductal gray resting state functional connectivity in migraine and the modulation effect of treatment. SCI REP-UK 2016;6

56. Wang Y, Zheng Y, Qu S, et al. Cerebral Targeting of Acupuncture at Combined Acupoints in Treating Essential Hypertension: An Rs-fMRI Study and Curative Effect Evidence. EVID-BASED COMPL ALT 2016;2016:1-12

57. Bao C, Liu P, Liu H, et al. Different brain responses to electro-acupuncture and moxibustion treatment in patients with Crohn’s disease. SCI REP-UK 2016;6

58. Bian Y, He X, Hu S, et al. Functional Connectivity Modulation by Acupuncture in Patients with Bell’s Palsy. EVID-BASED COMPL ALT 2016;2016:1-10

59. Zheng Y, Zhang J, Wang Y, et al. Acupuncture Decreases Blood Pressure Related to Hypothalamus Functional Connectivity with Frontal Lobe, Cerebellum, and Insula: A Study of Instantaneous and Short-Term Acupuncture Treatment in Essential Hypertension. EVID-BASED COMPL ALT 2016;2016:1-10

60. Wang X, Wang Z, Liu J, et al. Repeated acupuncture treatments modulate amygdala resting state functional connectivity of depressive patients. Neuroimage Clin 2016;12:746-752

61. Deng D, Liao H, Duan G, et al. Modulation of the Default Mode Network in First-Episode, Drug-Naïve Major Depressive Disorder via Acupuncture at Baihui (GV20) Acupoint. FRONT HUM NEUROSCI 2016;10

62. Li Y, Wang Y, Liao C, Huang W, Wu P. Longitudinal Brain Functional Connectivity Changes of the Cortical Motor-Related Network in Subcortical Stroke Patients with Acupuncture Treatment. NEURAL PLAST 2017;2017:1-9

63. Ning Y, Li K, Fu C, et al. Enhanced Functional Connectivity between the Bilateral Primary Motor Cortices after Acupuncture at Yanglingquan (GB34) in Right-Hemispheric Subcortical Stroke Patients: A Resting-State fMRI Study. FRONT HUM NEUROSCI 2017;11

64. Chang J, Zhang H, Tan Z, et al. Effect of electroacupuncture in patients with post-stroke motor aphasia. WIEN KLIN WOCHENSCHR 2017;129:102-109

65. Fu C, Li K, Ning Y, et al. Altered effective connectivity of resting state networks by acupuncture stimulation in stroke patients with left hemiplegia. MEDICINE 2017;96:e8897

66. Li Z, Zeng F, Yin T, et al. Acupuncture modulates the abnormal brainstem activity in migraine without aura patients. Neuroimage Clin 2017;15:367-375

67. Wang Z, Wang X, Liu J, et al. Acupuncture treatment modulates the corticostriatal reward circuitry in major depressive disorder. J PSYCHIATR RES 2017;84:18-26

68. Li Z, Zeng F, Yin T, et al. Acupuncture modulates the abnormal brainstem activity in migraine without aura patients. Neuroimage Clin 2017;15:367-375

69. Bao C, Wang D, Liu P, et al. Effect of Electro-Acupuncture and Moxibustion on Brain Connectivity in Patients with Crohn’s Disease: A Resting-State fMRI Study. FRONT HUM NEUROSCI 2017;11

70. Tan TT, Wang D, Huang JK, et al. Modulatory effects of acupuncture on brain networks in mild cognitive impairment patients. NEURAL REGEN RES 2017;12:250-258

71. Makary MM, Lee J, Lee E, et al. Phantom Acupuncture Induces Placebo Credibility and Vicarious Sensations: A Parallel fMRI Study of Low Back Pain Patients. Sci Rep 2018;8:930

72. Gollub RL, Kirsch I, Maleki N, et al. A Functional Neuroimaging Study of Expectancy Effects on Pain Response in Patients With Knee Osteoarthritis. The Journal of Pain 2018;19:515-527

73. Yeo S, van den Noort M, Bosch P, Lim S. A study of the effects of 8-week acupuncture treatment on patients with Parkinsonʼs disease. MEDICINE 2018;97:e13434

74. Li Z, Chen J, Cheng J, et al. Acupuncture Modulates the Cerebello-Thalamo-Cortical Circuit and Cognitive Brain Regions in Patients of Parkinson's Disease With Tremor. FRONT AGING NEUROSCI 2018;10

75. Pang Y, Liu H, Duan G, et al. Altered Brain Regional Homogeneity Following Electro-Acupuncture Stimulation at Sanyinjiao (SP6) in Women With Premenstrual Syndrome. FRONT HUM NEUROSCI 2018;12

76. Zhao J, Lu J, Yin X, et al. Comparison of Electroacupuncture and Mild-Warm Moxibustion on Brain-Gut Function in Patients with Constipation-Predominant Irritable Bowel Syndrome: A Randomized Controlled Trial. CHIN J INTEGR MED 2018;24:328-335

77. Zhang S, Wang X, Yan C, et al. Different mechanisms of contralateral- or ipsilateral-acupuncture to modulate the brain activity in patients with unilateral chronic shoulder pain: a pilot fMRI study. 2018;Volume 11:505-514

78. Kong J, Wang Z, Leiser J, et al. Enhancing treatment of osteoarthritis knee pain by boosting expectancy: A functional neuroimaging study. NeuroImage: Clinical 2018;18:325-334

79. Zeng Y, Cheng ASK, Song T, et al. Effects of Acupuncture on Cancer-Related Cognitive Impairment in Chinese Gynecological Cancer Patients: A Pilot Cohort Study. INTEGR CANCER THER 2018;17:737-746

80. Zheng W, Su Z, Liu X, et al. Modulation of functional activity and connectivity by acupuncture in patients with Alzheimer disease as measured by resting-state fMRI. PLOS ONE 2018;13:e196933

81. Wu P, Zhou Y, Liao C, et al. Structural Changes Induced by Acupuncture in the Recovering Brain after Ischemic Stroke. EVID-BASED COMPL ALT 2018;2018:1-8

82. Lee J, Eun S, Kim J, Lee J, Park K. Differential Influence of Acupuncture Somatosensory and Cognitive/Affective Components on Functional Brain Connectivity and Pain Reduction During Low Back Pain State. FRONT NEUROSCI-SWITZ 2019;13

83. Tu Y, Ortiz A, Gollub RL, et al. Multivariate resting-state functional connectivity predicts responses to real and sham acupuncture treatment in chronic low back pain. NeuroImage: Clinical 2019;23:101885

84. Xiang A, Yu Y, Jia X, et al. <p>The low-frequency BOLD signal oscillation response in the insular associated to immediate analgesia of ankle acupuncture in patients with chronic low back pain</p>. 2019;Volume 12:841-850

85. Han X, Bai L, Sun C, et al. Acupuncture Enhances Communication between Cortices with Damaged White Matters in Poststroke Motor Impairment. EVID-BASED COMPL ALT 2019;2019:1-11

86. Yu S, Lin S, Tsai C, et al. Acupuncture Effect and Mechanism for Treating Pain in Patients With Parkinson's Disease. FRONT NEUROL 2019;10

87. Zou Y, Tang W, Li X, Xu M, Li J. Acupuncture Reversible Effects on Altered Default Mode Network of Chronic Migraine Accompanied with Clinical Symptom Relief. NEURAL PLAST 2019;2019:1-10

88. Su C, Lu Q, Liu H, Luo C, Zhou Y. Amplitude changes in low frequency fluctuation in brains of primary dysmenorrheal treated by acupuncture: a resting-state fMRI study. INT J CLIN EXP MED 2019;12:8950-8956

89. Zhang J, Cai X, Wang Y, et al. Different Brain Activation after Acupuncture at Combined Acupoints and Single Acupoint in Hypertension Patients: An Rs-fMRI Study Based on ReHo Analysis. EVID-BASED COMPL ALT 2019;2019:1-10

90. Wang Y, Liu Z, Chen F, et al. Effects of acupuncture on craving after tobacco cessation: a resting-state fMRI study based on the fractional amplitude of low-frequency fluctuation. QUANT IMAG MED SURG 2019;9:1118-1125

91. Duan G, He Q, Pang Y, et al. Altered amygdala resting-state functional connectivity following acupuncture stimulation at BaiHui (GV20) in first-episode drug-Naïve major depressive disorder. BRAIN IMAGING BEHAV 2019

92. Yu S, Ortiz A, Gollub RL, et al. Acupuncture Treatment Modulates the Connectivity of Key Regions of the Descending Pain Modulation and Reward Systems in Patients with Chronic Low Back Pain. J CLIN MED 2020;9:1719

93. Kim H, Mawla I, Lee J, et al. Reduced tactile acuity in chronic low back pain is linked with structural neuroplasticity in primary somatosensory cortex and is modulated by acupuncture therapy. NEUROIMAGE 2020;217:116899

94. Chen S, Cai D, Chen J, Yang H, Liu L. Altered Brain Regional Homogeneity Following Contralateral Acupuncture at Quchi (LI 11) and Zusanli (ST 36) in Ischemic Stroke Patients with Left Hemiplegia: An fMRI Study. CHIN J INTEGR MED 2020;26:20-25

95. Han X, Jin H, Li K, et al. Acupuncture Modulates Disrupted Whole-Brain Network after Ischemic Stroke: Evidence Based on Graph Theory Analysis. NEURAL PLAST 2020;2020:1-10

96. Ren Y, Xu M, von Deneen KM, et al. Acute and long-term effects of electroacupuncture alter frontal and insular cortex activity and functional connectivity during resting state. Psychiatry Research: Neuroimaging 2020;298:111047

97. Liu CH, Yeh TC, Kung YY, et al. Changes in resting‐state functional connectivity in nonacute sciatica with acupuncture modulation: A preliminary study. BRAIN BEHAV 2020;10

98. Yan C, Huo J, Wang X, et al. Different Degree Centrality Changes in the Brain after Acupuncture on Contralateral or Ipsilateral Acupoint in Patients with Chronic Shoulder Pain: A Resting-State fMRI Study. NEURAL PLAST 2020;2020:1-11

99. Wang Y, Li T, Ha L, et al. Effectiveness and cerebral responses of multi-points acupuncture for primary insomnia: a preliminary randomized clinical trial and fMRI study. BMC Complementary Medicine and Therapies 2020;20

100. Zhang J, Lyu T, Yang Y, et al. Acupuncture at LR3 and KI3 shows a control effect on essential hypertension and targeted action on cerebral regions related to blood pressure regulation: a resting state functional magnetic resonance imaging study. ACUPUNCT MED 2020:974677030

101. Mawla I, Ichesco E, Zöllner HJ, et al. Greater Somatosensory Afference with Acupuncture Increases Primary Somatosensory Connectivity and Alleviates Fibromyalgia Pain via Insular GABA: A Randomized Neuroimaging Trial. ARTHRITIS RHEUMATOL 2020

102. Shi XH, Wang YK, Li T, et al. Gender‐related difference in altered fractional amplitude of low‐frequency fluctuations after electroacupuncture on primary insomnia patients: A resting‐state fMRI study. BRAIN BEHAV 2020

103. Li Z, Zhou J, Cheng S, et al. Cerebral fractional amplitude of low-frequency fluctuations may predict headache intensity improvement following acupuncture treatment in migraine patients. J TRADIT CHIN MED 2020;40:1041-1051

104. Ma K, Liu Y, Shao W, et al. Brain Functional Interaction of Acupuncture Effects in Diarrhea-Dominant Irritable Bowel Syndrome. FRONT NEUROSCI-SWITZ 2020;14

105. Wang Y, Qin Y, Li H, et al. The Modulation of Reward and Habit Systems by Acupuncture in Adolescents with Internet Addiction. NEURAL PLAST 2020;2020:1-9

106. Pang Y, Liao H, Duan G, et al. Regulated aberrant amygdala functional connectivity in premenstrual syndrome via electro-acupuncture stimulation at sanyinjiao acupointSP6. GYNECOL ENDOCRINOL 2020:1-5

107. Sun R, He Z, Ma P, et al. The participation of basolateral amygdala in the efficacy of acupuncture with deqi treating for functional dyspepsia. BRAIN IMAGING BEHAV 2020
